# Body Image and Its Associated Factors among People Living with HIV: A Scoping Review and Implications for Integrated Care

**DOI:** 10.1007/s10461-025-04991-6

**Published:** 2025-12-23

**Authors:** Atena Pasha, Mohammad Jahanaray, Xiaoming Li, Shan Qiao

**Affiliations:** 1 Department of Health Promotion, Education and Behavior, Arnold School of Public Health, University of South Carolina, Columbia, SC USA; 2 South Carolina SmartState Center for Healthcare Quality, Arnold School of Public Health, University of South Carolina, Columbia, SC USA; 3 School of Education, Virginia Commonwealth University, Richmond, VA USA

**Keywords:** HIV/AIDS, People living with HIV, Body image, Mental health, Scoping review.

## Abstract

People living with HIV (PLWH) face unique biopsychosocial challenges due to both infection and antiretroviral therapy (ART), one of which is body image disruption. This study comprehensively reviewed relevant studies to explore body image issues, identify associated factors, and describe assessment methods and interventions targeting body image in this population. Guided by the PRISMA, a thorough search of PsycINFO, PubMed, Embase, and Web of Science was conducted in November 2024, using search terms relevant to HIV and Body image. NIH Study Quality Assessment Tools were used to assess the quality of the included studies, and a narrative synthesis was conducted to identify common themes, including definitions of body image, associated factors, measurement instruments, and interventions targeting body image among PLWH. From 2197 publications, 26 studies met the inclusion criteria. The majority of studies demonstrated an association between negative body image and psychosocial factors, including depression, anxiety, social withdrawal, and substance use. Body image dissatisfaction was also associated with physical health factors such as lipodystrophy. Sixteen distinct body image measurement tools were used across studies. Cognitive Behavioral Therapy for Body Image and Self-Care (CBT-BISC) was the only target intervention that showed effectiveness in mitigating body image disturbance and improving ART adherence among PLWH. This is the first comprehensive scoping review to exclusively consider body image, associated factors, measurements, and target interventions among PLWH, which highlighted the need for comprehensive, culturally sensitive interventions that address both the physical and psychological dimensions of body image concerns.

**Supplementary Information:**

The online version contains supplementary material available at 10.1007/s10461-025-04991-6.

## Introduction

According to the World Health Organization (WHO), HIV remains a major global public health issue, with an estimated 39.9 million people living with the virus by 2024 and approximately 42.3 million deaths. While there is currently no cure for HIV, access to effective prevention, diagnosis, treatment, and care has transformed HIV into a manageable chronic health condition, allowing people living with HIV (PLWH) to enjoy long and healthy lives [1]. However, PLWH often encounter challenges related to the physical side effects of both HIV infection and antiretroviral therapy (ART), as well as psychological and emotional responses to these changes, such as body image [2, 3]. Body image is a multidimensional construct conceptualized as an individual’s subjective perceptions, thoughts, and feelings about their body or specific body parts [4]. These body image concerns may arise with or without objective physical changes and can be shaped by internal experiences and external social influences. In PLWH, such concerns may be amplified by the physical effects of the virus or ART, as well as stigma and other psychosocial stressors [5].

The perception of body image among PLWH can undergo significant changes over time [6, 7]. Following diagnosis, individuals may experience a profound impact on their self-concept, feeling invaded by the virus and now living with a chronic condition [6]. As the infection progresses, other changes may occur, either caused by the virus itself or by the side effects of ART, requiring ongoing monitoring. Additionally, interactions with healthcare providers and social relationships may influence an individual’s self-image as a chronic patient [7]. The stigma associated with receiving a diagnosis of HIV can also result in a variety of detrimental emotional and physical outcomes [8, 9]. It might influence individuals’ perceptions of their own bodies, highlighting the significance of monitoring any alterations in body image that may arise during treatment [10–12].

Social, physiological, and psychological factors, including peers, age, gender, and culture, shape individuals’ perceptions of their bodies [13]. Changes in a person’s body due to illness or other factors can deeply impact their sense of self [14, 15]. Individuals with disabilities, chronic diseases, or changes in appearance are particularly impacted by the unique social context surrounding these conditions, which can lead to distinct reactions in social relationships and self-perception [16–18]. These changes can profoundly impact a person’s body image, encompassing their mental representation of their body identity, including self-perceptions and attitudes toward bodily appearance and function [13, 15]. Moreover, studies indicate that an individual’s perception of their body image can significantly impact their adherence to ART medication among PLWH. Specifically, a negative body image has been correlated with non-adherence to medication [19]and a decrease in retention in care [20]and social isolation [21].

However, limited research has focused on understanding the psychological and social factors associated with body image concerns among PLWH. There is so far no comprehensive overview of body image issues related to HIV infection and associated factors. Additionally, there is no standardized measure to fully capture the concept of body image, although various methods, such as questionnaires and figure rating scales, have been used to assess body image. There is also a lack of interventions that address body image concerns among PLWH. Given the limited data on this topic and the demonstrated links between poor body image and negative clinical outcomes in PLWH, it is crucial to conduct a scoping review of existing literature regarding body image, associated biopsychosocial factors, body image measurement instruments, and interventions targeting body image among PLWH.

The current study aims to comprehensively review existing evidence from quantitative studies and randomized controlled trials (RCTs) on body image among PLWH. This is the first study of its kind to be conducted exclusively with PLWH that considered assessment tools and psychological interventions, which can be critically important for directing the distribution of resources and implementing various health programs to address physical, psychological, and social aspects of care for this population.

## Methods

### Study Design

This scoping review adhered to the preferred items for systematic reviews (PRISMA 2020) [22], ensuring the study’s rigor and reproducibility. The protocol was peer-reviewed and pre-registered on the Prospero website with ID number CRD42024530076. This scoping review did not involve primary data collection. All data were extracted from previously published studies that reported ethical approvals and participant consent, where applicable. The search methodology and related steps are presented below.

## Search Strategy

In November 2024, a comprehensive search across multiple electronic databases was conducted, including PsycINFO, PubMed, Embase, and Web of Science. This rigorous search strategy, which aimed at selecting the most relevant and eligible publications, was initially based on preselected keywords and phrases. The keywords were a series of combinations of terms related to Body Image (e.g., “Body Dissatisfaction,” “Body Image Disturbance,” “Body Concern,” “Body Satisfaction”) and HIV (e.g., “HIV,” “HIV/AIDS.”) (see Supplementary Material).

## Eligibility Criteria

The existing articles were assessed for eligibility based on the following criteria: (1) They should be empirical studies in English and published in peer-reviewed journals; (2) They should focus on body image in PLWH; and (3) They should report measures of one or more dimensions of body image. Additionally, there were no limitations on the age, gender, or ethnicity of the study participants.

This review excluded the following publications in accordance with its objectives: (1) studies published in a language other than English; or (2) studies that only contain qualitative studies, commentaries, study protocols, literature reviews, conceptual papers, and conference abstracts.

## Search Process

A two-part search strategy was used by two independent researchers to identify studies that were eligible for inclusion. First, we searched electronic bibliographic databases for published studies using a string of preselected keywords and phrases. Second, we searched the reference lists of records that met the criteria for inclusion in the review and the reference lists of relevant, previously published review articles for other records that may be eligible for inclusion. The Rayyan cloud-based platform [23] was used to manage the review process from the initial screening of records through to the selection of studies that were eligible for inclusion.

**Fig. 1:**
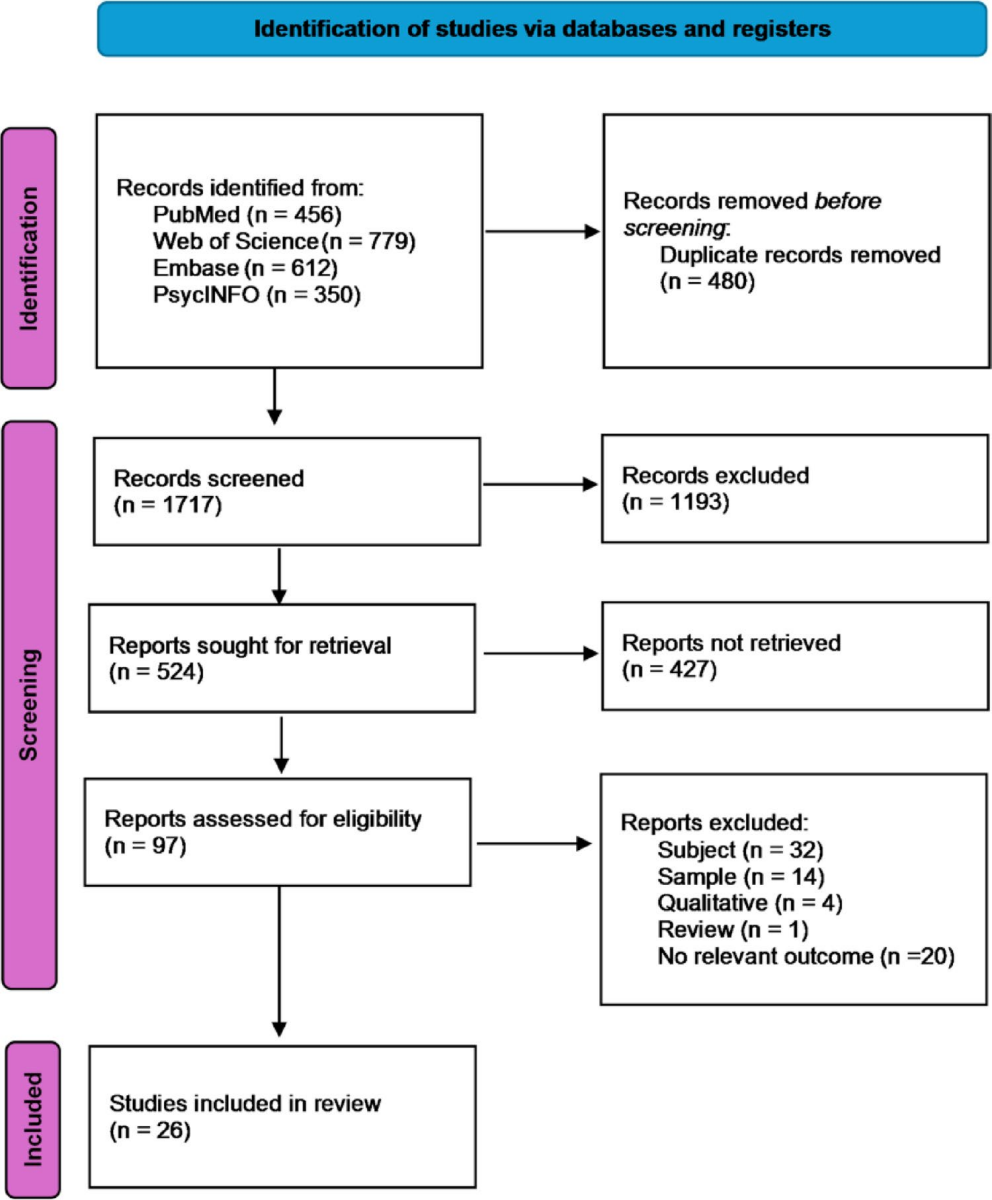
Flow diagram of the literature search and articles selection (adapted from PRISMA 2020 guidelines for systematic reviews)

Following the title and abstract screening, the full texts of potentially relevant articles were retrieved and reviewed for eligibility based on the pre-established inclusion and exclusion criteria (see Fig. 1). Any discrepancies were resolved through discussion. In cases where the agreement could not be reached, a third researcher was involved to adjudicate. This multi-stage review process ensured that eligible studies were systematically identified and included in the final analysis.

## Time Period

All eligible studies published from 1 January 2000 to 24 November 2024 were included. The period from early 2000 to the present marks an era of large-scale ART coverage.

### Quality Assessment

NIH Study Quality Assessment Tools were used to assess the quality of the included studies [24]. The Quality Assessment Tool for Observational Cohort and Cross-Sectional Studies was used for cross-sectional studies. Meanwhile, the Quality Assessment of Controlled Intervention Studies was used for Randomized Controlled Trial (RCT) studies.

Each question/dimension of the NIH tools was labeled with the following: “Not Reported (NR)” option used for studies that could potentially be answered “Yes” but the study did not report the required information; for instance, question 3 in the cross-sectional tool asks about the participation rate of eligible persons if a study only reported the number of participants but not the number of all of those who were approached initially this question would be “NR”. The “Cannot Determine (CD)” option is for those questions that are hard to answer, whether the study addresses them or not, because of lack of clarity. The “Not Applicable (NA)” option was used when one of the instrument’s criteria could not be evaluated due to the type of study (such as a cross-sectional design). The risk of bias/methodological quality of our included articles was assessed by two independent coders. Any discrepancies in the assessments were resolved through discussion among the researchers. Each study’s quality was labeled as each of the following:” Poor,” “Fair,” or “Good.”

## Data Extraction and Analysis

Data extraction was carried out using a standardized data extraction form designed to capture key study characteristics and findings. For each included study, the following information was extracted: author(s), year of publication, study design, sample size, population characteristics (e.g., age, gender, occupation), the body image definition and measurement tools used, key findings and associated factors related to body image, any reported interventions aimed improving body image among PLWH, limitation and future suggestion of included studies.

Two independent researchers extracted data from the included studies. All extracted data were recorded in a shared database to ensure consistency and accuracy. Any discrepancies in data extraction were resolved through discussion among the researchers.

## Data Synthesis

A narrative synthesis was conducted to identify common themes, including definitions of body image, associated factors, measurement instruments, and interventions targeting body image among PLWH. These themes informed the analysis by organizing and interpreting the extracted data, addressing the review’s research questions, and providing insights into the factors associated with body image. Additionally, the synthesis guided the categorization of measurement instruments and interventions, facilitating a comprehensive understanding of the methodologies and practices reported in the studies.

## Results

### Quality Assessment

Table 1 shows the quality assessment in this review. In the quality assessment of observational cohort and cross-sectional studies, several failed to meet specific criteria, impacting their overall robustness and validity. Notably, 16 studies (approximately 61.5%) did not report or deemed inapplicable the criteria related to participation rates of eligible persons (Q3), and three studies failed to have clear inclusion/exclusion criteria and uniformity in recruitment from similar populations (Q4). Similarly, 18 studies lacked sample size justification or discussion on statistical power (Q5), while 20 studies failed to measure exposures prior to outcomes (Q6). Based on the NIH tool description, some studies, like cross-sectional studies, would get “0” because of their study design type. Blinding of outcome assessors and loss to follow-up protocols were also inadequately reported or not applicable in all studies and 22 studies, respectively (Q12 and Q13). Lastly, seven studies did not adequately measure and adjust for key confounders (Q14), which is critical for isolating the exposure effects from other variables. It is important to note that most of our studies assessed the relationship between their variables of interest at a singular point in time. Consequently, responses to questions 6, 7, 10, 12, and 13 for nearly all these studies would correspondingly be “No” or “Not Applicable.” Therefore, the overall quality assessment of these studies was conducted based on eligible questions, intentionally excluding the non-eligible questions from the comprehensive evaluation.

**Table 1:** Quality assessment of included studies

Authors	Q1	Q2	Q3	Q4	Q5	Q6	Q7	Q8	Q9	Q10	Q11	Q12	Q13	Q14	Total Score	Decision
Blashill et al. [35]	Y	Y	NR	NR	N	NA	NA	Y	Y	NA	Y	NA	NA	N	5	fair
Lima et al. [37]	Y	Y	Y	Y	Y	N	N	Y	Y	NA	Y	NA	NA	Y	9	good
Fingeret et al. [27]	Y	Y	Y	Y	N	Y	Y	Y	Y	N	Y	NA	Y	Y	11	good
Junior et al. [38]	Y	Y	NR	Y	Y	N	N	Y	Y	NA	Y	NA	NA	Y	8	good
Corless et al. [34]	Y	Y	NR	Y	N	N	N	Y	Y	NA	Y	NA	NA	N	6	fair
Lamb et al. [30]	Y	Y	Y	Y	Y	Y	NR	NR	Y	Y	Y	NR	Y	CD	10	good
Gholizadeh et al. [42]	Y	Y	NR	Y	N	N	N	NA	Y	NA	Y	NA	NA	Y	6	good
Blashill et al. [20]	Y	Y	NR	N	N	N	N	Y	Y	NA	Y	NA	NA	Y	6	good
C.Martins et al. [11]	Y	Y	NR	Y	Y	N	N	Y	Y	NA	Y	NA	NA	Y	8	good
Raggio et al. [5]	Y	Y	NR	Y	N	N	N	Y	Y	NA	Y	NA	NA	Y	7	good
Klimek et al. [28]	Y	Y	NR	Y	Y	Y	Y	Y	Y	Y	Y	CD	Y	Y	12	good
Blashill et al. [31]	Y	Y	Y	Y	Y	Y	Y	NR	Y	Y	Y	NR	Y	Y	12	good
Blashill et al. [40]	Y	Y	NR	Y	N	N	N	Y	Y	NA	Y	NA	NA	Y	7	good
Zanlorenci et al. [36]	Y	Y	Y	Y	Y	N	N	Y	Y	N	Y	NA	NA	Y	9	good
Huang et al. [39]	Y	Y	Y	Y	N	N	N	N	Y	N	Y	NA	NA	Y	6	good
Guaraldi et al. [46]	Y	Y	Y	Y	N	N	NA	N	Y	NA	Y	NA	NA	N	6	fair
Luzi et al. [45]	Y	Y	Y	Y	N	N	N	Y	Y	NA	Y	NA	NA	Y	8	good
M.Martins et al. [11]	Y	Y	NR	Y	Y	NA	NA	NA	Y	NA	Y	NA	NA	NA	6	good
Palmer et al. [29]	Y	Y	NR	Y	N	Y	Y	Y	Y	CD	Y	NR	NA	Y	9	good
Sackey et al. [25]	Y	Y	N	Y	N	N	N	Y	Y	NA	Y	NA	NA	Y	7	fair
Sharma et al. [26]	Y	Y	NR	Y	N	NA	NA	Y	Y	NA	Y	NA	NA	Y	7	good
Martinez et al. [32]	Y	Y	NR	Y	N	Y	NA	N	Y	Y	Y	NA	NA	Y	8	fair
Theodore et al. [41]	Y	Y	NR	Y	N	N	N	Y	Y	CD	Y	NA	NR	Y	7	fair
Wilkins et al. [44]	Y	Y	Y	Y	N	N	N	Y	Y	NA	Y	NA	NA	NA	7	good
Wu [33]	Y	Y	NR	Y	N	N	N	NA	Y	NA	Y	NA	NA	NA	5	good
Soares et al. [43]	Y	Y	Y	N	N	N	N	Y	Y	NA	NA	NA	NA	Y	6	good

### Characteristics of Included Studies

 As shown in Tables 2, 26 studies out of the initial 2197 publications were included in the final review, which were conducted between 2004 and 2024, with the majority of the studies being carried out in the United States (17 studies) and Brazil (6 studies), and others in Italy (2 studies) and Canada (1 study). The sample sizes across these studies ranged from as few as 10 participants in Sackey et al. [25]to 550 participants in Sharma et al. [26]; However, on average, the majority of the studies had around 150 participants. Recruitment sites included internet-based platforms such as discussion boards and listservs, outpatient clinics, primary care clinics, community health centers, general and specialized hospitals, and other settings such as AIDS service organizations and private offices. The majority of the studies were cross-sectional (15 of the 26 reviewed studies). Three studies employed secondary analyses [27–29], while RCTs [30, 31], psychometric study [11, 32], and mixed-methods approaches [25, 33]were less common, each represented by two studies. Other study methods used include a correlation analysis [34]and a survey study [35].

### Sociodemographic Characteristics of PLWH

Table 2summarized the sociodemographic characteristics of PLWH. Participants’ ages ranged from 8 to 65. Studies with the adult group (aged 18 and above) had a mean age of 44.6 years across the studies, with a range of mean ages from 37 to 55.2. For the non-adult group (aged under 18), the mean age across studies was 15.1 years, with a range of mean ages from 11.93 to 20.7 years. Zanlorenci et al. [36] reported the lowest mean age (11.93 ± 6.99), and Sharma et al. [26] reported the highest mean age (55.2 ± 4.5). Out of a total of 4,095 PLWH, 3062 were males, and 1033 were females. Gender distribution across the studies predominantly featured male participants, although several studies included substantial female representation [5, 12, 36–38]. However, 11 studies focused on only male participants [20, 25, 26, 28, 30, 31, 35, 39–42]. The racial composition of participants varied across studies, with a significant proportion of participants identifying as Caucasian (1149) and Black/African American (702), Hispanic/Latino (243), Asian (12), Native-American (10), Multiethnic (5), Native Hawaiian (1), and 232 categorized as Other.

**Table 2:** Sample characteristics of included papers

ID	Citation	Country	Design	Sample Size	Sample Characteristics	Recruitment site	Mode of HIV acquisition	Time of HIV diagnosis	Medication use	Comorbid Condition	BMI
1	Blashill & Vander wal [35]	United States	Cross-sectional	254 gay men with HIV	Age: 42.27 ± 13.38; man;85.4% Caucasian,5.1% Hispanic/Latino, 4.3% Black/African, 2.4% Asian, 1.6% Multiethnic, 0.8% Native American, 0.4% Hawaiian Native	Various gay and HIV/AIDS-related Internet-based discussion boards and listserves	Not Reported	Most diagnosed with HIV more than 10 years ago (*n* = 78, 56.1%), 6 to 10 years (*n* = 20, 14.4%), 2 to 6 years (*n* = 28, 20.2%), or less than 1 to 2 years (*n* = 13, 9.3%).	123 (88.5%) were taking medication to treat their conditions at the time of the study.	Not Reported	26.37 ± 6.34
2	Lima et al. [37]	Brazil	Cross-sectional	111 PLWH	Age: 13 (10–15 years).	Outpatient clinic	Mother-to-child transmission	Not Reported	Not Reported	Not Reported	18.52 ± 2.62
3	Fingeret et al. [27]	United States	Secondary data analysis	77 PLWH	Age: 43.48 +_ 7.77 (27–64); 60 men, 17 women; 75% African American, 20% Caucasian, 5% Hispanic	Primary care clinic within a large, inner-city HIV/AIDS center	32.5% male-to-male sexual contact, 39% heterosexual contact, 22% injection drug use, 6.5% other	Not Reported	Not Reported	Not Reported	Not Reported
4	Junior et al. [38]	Brazil	Cross-sectional	65 HIV-children and adolescents	Age: 12.2 (8–15 years). 30 Male and 35 Female	Federal University of Santa Catarina	Mother-to-child transmission	Not Reported	83.1% were using ART	Not Reported	17.92 ± 2.65
5	Corless et al. [34]	United States	Correlational study	40 HIV-patients	Age: 39.5 23 Male, 17 Female	Primary care clinic	Not Reported	Men = 9.2 years, Women = 7.2 years	Not Reported	Not Reported	Not Reported
6	Lamb et al. [30]	United States	Randomized controlled trial (RCT)	44 sexual minority men living with HIV	Age: 46 (SD = 11); man; 64% White, 34% African American/Black, 23% Hispanic/Latino	Community health center in Boston	Not Reported	Not Reported	All taking ART	42% with at least one psychiatric condition, 73% with two or more, 68% BDD, 46% MDD, 34% GAD	27 ± 4.9
7	Gholizadeh et al. [42]	United States	Cross-sectional	105 sexual minority men living with HIV who reported having sex with a man within the previous 12 months.	Age: mean of 37 with a range of 18–65 years	General Hospital Infectious Disease Clinic and a community health center	Not Reported	13.15 years (± 8.2)	All taking ART	Not Reported	27.96 ± 4.67
8	Blashill & Vander Wal [20]	United States	Cross-sectional	124 gay and bisexual men living with HIV	Age: 47.94 (SD = 9.51); man; 85.5% Caucasian, 7.3% Hispanic/Latino, 4.8% Black/African American, 0.8% Multiethnic, 0.8% Asian, 0.80% Native-American	Various HIV/AIDS related Internet-based discussion boards and listservs	Not Reported	1 year (1.6%) 1–2 years (4.0%) 2–4 years (10.5%) 4–6 years (8.9%) 6–8 years (11.3%) 8–10 years (4.8%) 10 years (58.9%)	HAART non-adherence for 10.57 years ± 1.72 for all participants	Not Reported	25.60 ± 5
9	C.Martins et al. [12]	Brazil	Cross-sectional	412 PLWH	Age: 45.2 (SD = 16.3); 60.8% women, 39.2% male	A follow-up service (STD/AIDS Specialized Service-SAE)	Not Reported	Not Reported	90.20% used HAART over the past year	Not Reported	Not Reported
10	Raggio et al.[5]	United States	Cross- sectional	63 women living with HIV	Age: 51 (45–79); women; 58.7% African American/Black, 24% Caucasian, 2% American Indian/Alaska Native, 16% other, 11% Hispanic/Latina	A private office in the principal investigator’s hospital	Not Reported	14.1 ± 7.3	95.10% used ART at the time of survey completion	70% depression, 11% anxiety	Not Reported
11	Klimek et al. [28]	United States	Secondary data analysis	22 sexual minority men living with HIV and body image disturbance (43 gay, 1 bisexual)	Age: 46 (SD = 11) (18–65 years); 63.6% White, 34.1% Black, 4.5% Native American, 4.5% other	Fenway Health, a community health center in Boston	Not Reported	Not Reported	Used ART for the past two months	68.2% BDD, 18.2% ED, 95.5% at least one psychiatric diagnosis	Not Reported
12	Blashill et al. [31]	United States	RCT	44 sexual minority men living with HIV who reported elevated appearance concerns	Age: 46.18 (SD = 11.03) (18–65); man; 34.10% African American/Black, 63.60% White, 4.5% Native American, 4.5% other, 22.7% Hispanic/Latino	Fenway Health, a community health center	Not Reported	Not Reported	Used ART for the past two months	95.5% at least one psychiatric diagnosis, 72.72% two or more diagnoses, 68.20% Body dysmorphic disorder, 45.50% Major depressive disorder, 34.10% Generalized anxiety disorder, 27.30% Dysthymic disorder, 20.50% Substance dependence, 18.20% eating disorder, 15.90% Social anxiety disorder, 15.90% Alcohol dependence	27.3 ± 4.88
13	Blashill et al. [40]	United States	Cross- sectional	106 gay and bisexual men living with HIV	Age: 47.5 (SD = 7.8) (18–65); man; 12% Hispanic/Latino, 73% White, 23% Black/African American, 3% Native American, 4% other, 1% Asian	General Hospital Infectious Disease Clinic, and Fenway Health, a community health center	Not Reported	13.1 ± 8.2	Used ART for the past two months	Depressive symptoms (mean CES-D ≈ 20); lipodystrophy symptoms	27.1 ± 4.6
14	Zanlorenci et al. [36]	Brazil	Cross-sectional	60 children and adolescents diagnosed with HIV	Age: 11.93 (SD = 6.99) (8–15 years); 32 females; 28 males	Outpatient clinic of a regional HIV reference center	Mother-to-child transmission	Not Reported	83.33% were using ART	Lipoatrophy	17.8 ± 2.71
15	Huang et al. [3]	United States	Correlational study	110 men living with HIV	Age: 44 (38–50); man; 26% Hispanic, 8% Black, 59% White, 1% Asian, 6% other	An academic multidisciplinary adult HIV clinic	Not Reported	Not Reported	ART	43.6%−48.2% Lipodystrophy (self-reported vs. physician-rated); 26.4% depression and anxiety, 65.5% AIDS	Not Reported
16	Guaraldi et al. [46]	Italy	Cross- sectional	330 PLWH	Age: 41.5 (SD = 6.5); 26.74% female, 73.25% male	HIV outpatient clinic	Not Reported	Not Reported	All were on ART for at least 6 months	89.5% lipodystrophy	22.5 ± 3
17	Luzi et al. [45]	Italy	Cross- sectional	185 women living with HIV	Age: 42 (SD = 5); Females;	Routine HIV facility care	Not Reported	16 years for FSD and 13.8 years without FSD	86% are currently on ART at baseline	81% lipodystrophy, 6% diabetes, 11% menopause	Not reported
18	M.Martins et al. [11]	Brazil	Psychometric Study	400 Brazilian PLWH	Age: 41.12 (SD = 11.68) (18–78); 67.8% male, 35% female; 53.8% Caucasian, 9.3% Black, 43% other	An HIV/AIDS ambulatory	Not Reported	Not reported	All were on ART	51.8% Lipodystrophy	Not Reported
19	Palmer et al. [29]	Canada	Secondary data analysis	451 PLWH	Age: mean of 46. 24% female, 76% male; 33% Aboriginal, 67% Non-aboriginal	Centre for Excellence in HIV/AIDS (BCCfE)	Not Reported	Not Reported	90% were currently on ART	57% had depressive symptoms	Not Assessed
20	Sackey et al. [25]	United States	Mixed-Method	10 PLWH	Age: 54.95 (SD = 3.02); 72.5% Black, non-Hispanic, 12.5% White, non-Hispanic, 10% Hispanic/Latino, 2.5% Mixed, non-Hispanic	Gay Men’s Health Crisis (GMHC), a leading AIDS service organization	Not reported	Minimum years living with HIV was 18 years	93% were on ART	55% High cholesterol, 32.5% High blood pressure, 10% Type 2 diabetes, 20% Lipodystrophy, 27.5% Osteoporosis, 20% Opportunistic infection in last 5 years, 32.5% Arthritis, 20% CVD	25.25 ± 3.7
21	Sharma et al. [26]	United States	Cross-sectional	550 (322 PLWH) older men with or at risk for HIV infection	Age: 55.2 (SD = 4.5) (49–74); 12.4% White, 59% Black, 22.7% Hispanic, 5.9% other	Montefiore Medical Center	Not reported	11 years (7–15)	86.3% on ART	43.8% Depressive Symptoms: of HIV-infected men, 36% Erectile Dysfunction	Not Assessed
22	Martinez et al. [32]	United States	Cross-sectional	150 PLWH	Age: 42 (22–65); 78.9% male, 21.1% female; 51.7% Caucasian, 21.9% Latino, 13.9% African American, 2.6% Asian, 6.6% other	Outpatient clinic (a public hospital HIV clinic and a university hospital HIV clinic)	Not Reported	1 months-19 years	Not Reported	Not Required	Not Reported
23	Theodore et al. [41]	United States	Cross-sectional	42 Gay Bisexual Men living with HIV	Age: 37.5 (SD = 5.4); 47.61% White/Non-Hispanic, 40.47% Latino/Hispanic, 9.52% African American, 2.38% other	Recruited at a “gay beach” in South Florida	Not Reported	Not Reported	30 participants (71.4%) used HIV medication	Not Reported	Not Assessed
24	Wilkins et al. [44]	United States	Cross- sectional	119 Youths living with HIV	Age: 20.7 (SD = 1.98); 89 Male, 30 female. Caucasian: 5, Hispanic: 1, African American: 113	Infectious diseases clinic	83.21% behavioral, 16.78% perinatal	24.75 ± 5.36	110 participants (76.9%) were on ARVs	Not Reported	25.3 ± 6.1
25	Wu et al. [33]	United States	Mixed-Method	96 adults living with HIV	Age: 48.8 (SD = 8.3). 84 Male, 12 Female. Caucasian: 64, Asian: 1, Hispanic: 7, African American: 20	HIV Clinic	Not reported	Not reported	All had a history of ARV therapy	Facial lipoatrophy	Not Assessed
26	Soares et al. [43]	Brazil	Cross-sectional	231 adults living with HIV	Age: 40 (SD = 8.9). 154 Male, 77 Female	Immunodeficiencies Outpatient Clinic	Not Reported	Mean of 42 months.	72.3% were on ART, with 28.5% exposed to protease inhibitors (PIs)	40% had self-reported lipodystrophy	Not Assessed

The modes of HIV acquisition were not reported in most of the included articles, except for five studies, where four out of them mentioned Mother-to-child transmission [36–38, 43], and Fingeret et al. [27] reported that 32.5% acquired HIV through male-to-male sexual contact, 39% through heterosexual contact, and 22% via injection drug use. The duration since HIV diagnosis varied considerably among the studies. A notable portion of participants (56.1%) had been diagnosed with HIV for over a decade [5, 25, 26, 40, 42, 44, 45]. Other studies consisted of participants with shorter durations of diagnoses, with groups diagnosed between 6 and 10 years (14.4%), 2 to 6 years (20.2%), and less than 1 to 2 years (9.3%) [20, 34, 35]. The duration of diagnosis also varied by gender, with men having an average of 9.2 years and women 7.2 years.

Only 8 studies reported the type of medications or treatment that PLWH were on at the time of the study. The most commonly reported ART regimens included Nucleoside Reverse Transcriptase Inhibitors (NRTIs), Non-Nucleoside Reverse Transcriptase Inhibitors (NNRTIs), and Protease Inhibitors (PIs) [25, 34, 37, 38, 43–45].

### Body Image Dimensions and Measurement

The studies have focused on different indicators of body image. Fingeret et al. [27], Blashill and Vander Wal [20], Blashill and Vander Wal [35], Theodore et al. [41], and Gholizadeh et al. [42] concentrated on PLWH body dissatisfaction. While Blashill et al. [40], Blashill et al. [31], Lamb et al. [30], and Klimek et al. [28] delved into body image disturbance and its nuances in PLWH. Martinez et al. [32], Guaraldi et al. [46], Wilkins et al. [44], and Zanlorenci et al. [36] studied body image perception among PLWH. Luzi et al. [45], Raggio et al. [5], and Soares et al. [43] focused on PLWH body satisfaction. Negative body image among PLWH was assessed by Sharma et al. [26] and Palmer et al. [29]. Corless et al. [34], Huang et al. [39], Lima et al. [37], and Junior et al. [38] aimed at assessing overall body image. Other studies by Wu et al. [33], Sackey et al. [25], M. Martins. et al. [11], and C. Martins. et al. [12] explored Cognitive and Behavioral Aspects of PLWH Body Image Anxiety, Body Image Attitude, Body Image Distress and Discomfort, and self-perception of body image, respectively.

Among the 26 included studies, 16 different body image measures were utilized to assess body dissatisfaction, perception, and related psychological constructs among PLWH (see Table 3). The reliability of these measures, reported as Cronbach’s Alpha, varied across studies, indicating differences in internal consistency. The Assessment of Body Change Distress scale (ABCD) was one of the most frequently used instruments, appearing in Guaraldi et al. [46] (α = 0.94), Blashill et al. [40] (α = 0.84), and Wu et al. [33] (α = 0.98), also in Luzi et al. [45] and Raggio et al. [5], but with no reports on the internal consistencies. This scale demonstrated high reliability, making it a robust tool for evaluating body disturbance and satisfaction associated with ART-related physical changes. The Multidimensional Body-Self Relations Questionnaire (MBSRQ) was used in Blashill and Vander Wal [35] (α = 0.92) and Wilkins et al. [44] (α = 0.74). These findings suggest that the MBSRQ remains a reliable tool for assessing body image dissatisfaction and perception among PLWH.

The silhouette scale, commonly used to measure perceived body size, was employed in Lima et al. [37], Martins, C et al. [12], Junior et al. [38], and Zanlorenci et al. [36], but none of the studies reported the internal consistency. The reliability of this tool to assess body image perception among PLWH requires further exploration. The Body Image Scale (BIS), another commonly used measure, was utilized in Martinez et al. [32] (α = 0.91) and Fingeret et al. [27] (α = 0.88), indicating strong internal consistency. Additionally, the Body Dysmorphic Disorder modification of the Yale-Brown Obsessive-Compulsive Scale (BDD-YBOCS), which specifically assesses body dysmorphic concerns, was employed in Blashill et al. [31] (α = 0.93) and Lamb et al. [30] (α = 0.93).

Several other body image scales were used in individual studies, demonstrating varied but generally high reliability. The Medical Outcomes Study-HIV (MOS-HIV) instrument used in Corless et al. [34] showed a reliability score of 0.90. The Situational Multidimensional Body-Self Relations (MBS), used by Blashill and Vander [20], exhibited a Cronbach’s Alpha of 0.91, while the Male Body Attitudes Scale (MBAS) employed by Sackey et al. [25] showed a reliability coefficient of 0.79. Lastly, the Body Image Disturbance Questionnaire (BIDQ) was used in Klimek et al. [28] and the Body Image Quality of Life (BIQLI) and the Brazilian Portuguese version of the Derriford Appearance Scale 24 (DAS-24), used in M. Martins et al. [11], demonstrated high reliability (α = 0.96 and α = 94, respectively).

### Body Images, Correlates, and Consequences

As shown in Table 3, several studies reported high levels of body dissatisfaction and negative body image perceptions among PLWH related to physical changes induced by HIV and its treatment. C. Martines et al. [11] found that PLWH (74.3% of all participants, 100% of the patients with low weight and 89.5% of the obese patients) and participants with Negative self-perception body image were 2.88 (Adjusted Prevalence Ratio) times more likely to exhibit depressive symptoms (95% CI 1.59–5.20; *p*= 0.001), a finding that may reflect the intersection of HIV-related stigma and body image perceptions. Gholizadeh et al. [42] highlighted specific dimensions of body image, noting a mean appearance investment score of 3.61 (SD = 0.55; range = 2.33–4.75) and a body dissatisfaction score of 31.35 (SD = 8.17; range = 13.00–54.00), indicating considerable concerns related to appearance and dissatisfaction. Sharma et al. [26] differentiated between positive and negative body image, as 31% of participants reported a negative body image, with no significant difference between men who living with and without HIV, and among those with normal BMI, 24% reported negative body image, compared with 30% of overweight and 49% of obese men (*p* ≤ 0.001). Additionally, negative body image was significantly associated with several factors, including higher BMI, depressive symptomatology, erectile dysfunction, and poor self-rated health (AORs = 1.05 [1.01–1.09], 1.64 [1.08–2.48], 1.85 [1.23–2.80], and 2.07 [1.34–3.20], respectively).

**Table 3:** Body image measurement instruments and key findings

ID	Citation	Name of instrument	Instrument focus	Cronbach’s alpha	Findings
1	Blashill & Vander Wal [35]	MBSRQ	Body dissatisfaction	Appearance Evaluation: 0.92 Appearance Orientation: 0.88 Fitness/Health Evaluation: 0.83 Fitness/Health Orientation: 0.092 Illness Orientation: 0.76	Men in the AIDS group reported being less fit and in poorer health than men living with HIV. There were no significant differences between groups on appearance evaluation.
2	Lima et al. [37]	silhouette scale	Body image	Not Reported	Body Image dissatisfaction was linked to a desire to increase body size (38.6%), especially in males, and was associated with lower anthropometric measures like skinfolds and UAMA (Upper Arm Muscle Area), while gender, age, weight, BMI, and UAMA explained 42% of body image score variation.
3	Fingeret et al. [27]	BIS	Body image/appearance dissatisfaction	0.88	In PLWH, body image concerns were associated with depression, anxiety, stress, and lower social support, yet smoking cessation rates were highest at moderate concern levels and lowest at both high and low extremes, suggesting a unique curvilinear relationship not solely tied to weight concerns.
4	Junior et al. [38]	Silhouette scale	Body image	Not Reported	In children and adolescents living with HIV, body image dissatisfaction was prevalent, with 38.6% wanting to increase body size (notably males) and 40.7% of a control group wanting to reduce it. Among female PLWH, those desiring to reduce body weight had higher BMI and fat mass compared to those satisfied or wanting to increase it, while no significant differences were found in males across body image categories.
5	Corless et al. [34]	MOS-HIV	Body image (actual and ideal)	0.90	It found that weight change in men was related to better mental health, vitality, and quality of life scores. For women, weight change did not significantly correlate with these domains. Body image scores were higher for women than men, and there was no significant difference in body image between participants living with HIV and those with AIDS.
6	Lamb et al. [30]	BDD-YBOCS	Body image disturbance	0.84 at baseline to 0.93 at the 3-month follow-up	Participants assigned to CBT-BISC reported statistically significant reductions in body image disturbance post-intervention, which subsequently predicted changes in ART adherence from post-intervention to long-term follow-up. One pathway in which CBT-BISC positively impacts ART adherence was through reductions in body image disturbance.
7	Gholizadeh et al. [42]	The Muscle Dysmorphic Disorder Inventory (MDDI), Appearance Orientation subscale of the Multidimensional Body-Self Relations	Body dissatisfaction	0.72	A generalized linear model identified a significant interaction (b = 0.08 [95% CI: 0.01, 0.16], *p* = 0.033) such that when appearance investment was low, body dissatisfaction was associated with fewer condomless anal sex acts; when appearance investment was high, body dissatisfaction was associated with increased condomless anal sex.
8	Blashill & Vander Wal [20]	MBSRQ	Body dissatisfaction	0.91	The results of the moderated mediation models indicated that depression mediated the relationship between body dissatisfaction and HAART non-adherence for men with high levels of body dissatisfaction but not for those with moderate or low levels. Furthermore, depression also mediated the relationship between body dissatisfaction and HAART non-adherence among men who self-reported an AIDS diagnosis.
9	C. Martins et al. [ 12]	Silhouette scale	The self-perception of body image (SPBI)	Not Reported	Patients who use HAART are experiencing new challenges, such as metabolic and morphological body changes, which may affect self-perceived body image . Based on the results obtained, it can be concluded that negative self-perception of body image (NSPBI) in HIV-infected patients was associated with Nadir CD4, variations in BMI, particularly LW and obesity, self-perception of specific body changes and mental health (higher prevalence of depressive symptoms).
10	Raggio et al. [5]	The AIDS Clinical Trials Group Assessment of Body Change and Distress scale	Body satisfaction	Not Reported	Findings present a clear profile of the psychosocial and body image challenges associated with prevalent lipodystrophy among aging, actively treated Women living with HIV (WLWH). There were a persistent body image disturbance among patients over a decade after being diagnosed with HIV. Also, social support was not significantly associated with lipodystrophy severity or either of the prevalent lipodystrophy phenotypes in bivariate analyses. More than a third of women reported severe/diffuse lipodystrophy, defined herein as three or more body parts evidencing fat redistribution.
11	Klimek et al. [28]	BIDQ, BICSI	Body image disturbance, Body image coping skills	0.88 at baseline and 0.96 at follow-up	CBT-BISC significantly reduced body image disturbance and improved coping skills. Latent difference score mediation indicated that changes in acceptance and cognitive reappraisal significantly predicted body image disturbance changes.
12	Blashill et al. [31]	BDD-YBOCS, the Brown Assessment of Beliefs Scale (BABS)	Body image disturbance	BDD-YBOCS: 0.84 at baseline and 0.93 at the 3-month follow-up BIDQ: 0.88 at baseline and 0.96 at the 3-month follow-up.	The study demonstrated that CBT-BISC effectively reduces body image disturbance and depressive symptoms while improving adherence to HIV treatment among sexual minority men living with HIV. These improvements were substantial and maintained over the long term, indicating that integrated therapeutic approaches that address both mental health and physical care are crucial for this population.
13	Blashill et al. [40]	Body Change and Distress Questionnaire–Short Form (ABCD-SF), MBSRQ	Body image disturbance	0.84	Lipodystrophy severity and appearance orientation were associated with elevated body image disturbance. In turn, body image disturbance was related to poorer ART adherence and increased HIV sexual transmission risk behaviors, through the mechanisms of elevated depressive symptoms and poor condom use self-efficacy.
14	Zanlorenci et al. [36]	Silhouette scale	Body image perception	Not Reported	The results indicated that 53.13% of women and 53.57% of men were dissatisfied with their BI. Lower subscapular skinfold and higher calf skinfold values were associated with Body Image dissatisfaction in females. Pre-pubertal maturation stage, higher economic level, lower concentrations of CD4 + lymphocytes, lower viral load, lower level of physical activity, and longer time in front of the computer and/or video game were associated with Body Image dissatisfaction in males.
15	Huang et al. [39]	BIQLI, SIBID-S	Body image	Not Reported	Compared to men who denied lipodystrophy, men with self-reported lipodystrophy demonstrated poor body image. Physician-rated lipodystrophy was significantly associated with both body image subscale scores.
16	Guaraldi et al. [46]	ABCD	Body image perception	0.94	Preliminary evidence supports the reliability and validity of the Italian version of the ABCD in people living with HIV and lipodystrophy.
17	Luzi et al. [45]	Italian version of the ABCD	Body satisfaction	Not Reported	Desire, arousal, and satisfaction domains were associated with interference of body changes with habits, social life, and attitudinal aspects of body image. Lubrication and orgasm domains of the Female Sexual Function Index (FSFI) were associated with body image satisfaction. Lipodystrophy’s impact on body image was the sole risk factor associated with Female sexual dysfunction (FSD). This implies that changes in body composition and appearance, which can be significant in PLWH undergoing treatment, play a critical role in influencing sexual function.
18	M. Martins et al. [11]	Brazilian Portuguese version of DAS-24	Body image distress and discomfort	0.94	The Brazilian Portuguese version of DAS-24 seems to be a psychometrically sound scale for measuring body image distress for PLWH.
19	Palmer et al. S. [29]	BIS	Negative body image	Not Reported	Of 451 The Longitudinal Investigations into Supportive and Ancillary Health Services (LISA) participants, 47% reported negative body image. The adjusted multivariate analysis showed participants who reported high stigma in the presence of depressive symptoms were more likely to have negative body image compared to people reporting low stigma and no depressive symptoms. The estimated probability of a person having a positive body image without stigma or depression was 68%. When stigma alone was included, the probability dropped to 59%, and when depression was included alone, the probability dropped to 34%. Depressive symptoms and high stigma combined resulted in a probability of reporting positive body image of 27%.
20	Sackey et al. [25]	MBAS	Body image attitude	0.79	Participants reported challenges accessing healthy food and eating fruits and vegetables. Living with HIV/AIDS also influenced their food options, which in turn affected physical activity and body image.
21	Sharma et al. [26]	Self-reported	Negative body image	Not Reported	31% of participants reported negative body image, which was independently associated with increased BMI, self-rated fair/poor health, depression, and erectile dysfunction, but not HIV status.
22	Martinez et al. [32]	BIS	Body image perception	0.91	The BIS had good construct validity and was a highly reproducible measure of self-perception of body image in PLWH .
23	Theodore et al. [41]	BSQ	Body dissatisfaction	Not Reported	The study identified that body dissatisfaction explains a small but significant variance in methamphetamine use among gay and bisexual men living with HIVafter accounting for the effects of circuit party attendance and the use of alcohol and marijuana. This association highlights the psychological impact of body image issues on substance use behaviors within this demographic.
24	Wilkins et al. [44]	MBSRQ, Figure Rating Scale (FRS)	Body image perception	0.74	Results showed a normative global body image on the MBSRQ-Appearance Scales. Some subscale elevations were observed, including decreased interest in self-care and appearance, as well as concerns with individual body areas. Overall, youth reported a preference for their own body shape on the Figure Rating Scale; however, 41% of youth classified as ‘‘overweight’’ per CDC body mass index reported contentment with their current body size. Further, 47% of youth classified as ‘‘normal’’ weight desired to have a larger body size. Youth identified as men who have sex with men most often reported desiring larger body size.
25	Wu et al. [33]	ABCD, MOS-HIV	Cognitive and behavioural aspects of body image anxiety	0.98	The study found that the Facial Appearance Inventory (FAI) is a reliable and valid measure of appearance-related distress associated with HIV-related facial lipoatrophy. The FAI correlated more strongly with mental health than physical health, indicating the significant psychological impact of facial lipoatrophy. More severe lipoatrophy was associated with worse FAI scores, underscoring its negative effect on quality of life. Exploratory analyses suggested that individuals with lower educational attainment and those with higher Fitzpatrick skin types reported less perceived impairment on the FAI, suggesting variations in the perceived impact of lipoatrophy across different demographic groups.
26	Soares et al. [43]	Self-Reported	Body image satisfaction	Not Reported	The study found that 40% of participants experienced self-reported lipodystrophy, significantly affecting their body image, particularly through changes like fat loss in visible areas and increased waistlines. Notably, the use of lipid-lowering medications was positively correlated with improved self-perceptions of body image. Objective measurements via anthropometric and biochemical profiles were more effective than subjective reports, revealing that patients often perceived their body condition as worse than it was.

BMI was commonly included as a correlate of body image in the literature. While BMI reflects body composition, body image is a perceptual and psychological construct, shaped by individual and sociocultural factors. Therefore, BMI does not directly predict body image outcomes, and associations between the two were often weak, inconsistent, or even contradictory. For instance, a study conducted among youth living with HIV showed that those classified as overweight (41%) were often satisfied with their body size, while individuals with normal BMIs (47%) expressed a desire for a larger body [44]. This divergence may be due to reliance on self-reported BMI in the majority of the included studies, a method prone to misreporting and social desirability bias. These inconsistencies suggest that while BMI may influence or reflect body image concerns among PLWH, it should be interpreted cautiously, as it does not capture the subjective and multidimensional nature of body image.

Moreover, metabolic and morphological changes resulting from HIV and ART use have a profound impact on body image and overall well-being. Lipodystrophy is characterized by either partial or complete loss of fat in certain areas of the body, including the face and limbs, along with abnormal fat accumulation in other areas, such as the abdomen [47]. Lipoatrophy, on the other hand, specifically refers to the loss of subcutaneous fat, typically in the face and extremities, without the central fat accumulation seen in lipodystrophy [48]. Lipodystrophy and lipoatrophy both contribute to substantial psychological distress, social withdrawal, and a reluctance to disclose one’s HIV status due to fears of visible HIV-related stigma [46]​. The psychosocial burden of these physical changes is particularly pronounced in individuals with facial lipoatrophy, as they often report lower self-esteem and higher rates of social anxiety [33]​. Five studies reported the prevalence of lipodystrophy among participants, which ranged from 27.5% to 81%. Soares et al. [43] found that 40% (*N* = 98) of PLWH in their study reported self-perceived lipodystrophy. Further, biochemical and anthropometric measures showed significant correlations with body-image dissatisfaction, including body adiposity index (Spearman ρ = − 0.14, *p* = 0.036), percent fat by bioimpedance (Spearman ρ = − 0.14, *p* = 0.030), very-low-density lipoprotein ( Spearman ρ = − 0.14, *p* = 0.038), and triglycerides (Spearman ρ = − 0.13, *p* = 0.045), while triceps skinfold thickness correlated positively with better body-image perception (Spearman ρ = 0.14, *p* = 0.031). The study also noted a positive association between the use of lipid-lowering medications and self-perceived body image.

Several sociodemographic factors, including gender, minority status, and age, influence the way PLWH perceive their bodies, with notable variations across different subgroups. Women living with HIV consistently report higher levels of body dissatisfaction compared to men. C. Martins, et al. [12] found that 74.3% of patients exhibited negative self-perception of body image, with a higher prevalence among women (81.2%) compared to men (65.6%). Among men, sexual minority status emerged as a critical factor influencing body image concerns; while younger participants (18–40 years old) were twice as likely to report negative self-perception of body image (NSPBI) (Adjusted PR = 2.06, 95% CI [1.25–3.42], *p*= 0.005). Theodore et al. [41] found that HIV-positive gay and bisexual men exhibited high levels of body dissatisfaction, which was positively correlated with methamphetamine use (Pearson *r* = 0.41, *p* < 0.01). These findings suggest that body image concerns among sexual minority men may be linked to both internalized stigma and cultural expectations within LGBTQ + communities.

Body image concerns fluctuate across different age groups among PLWH, particularly in response to lipodystrophy and ART-associated weight changes. Wilkins et al. [44] examined body image in adolescents living with HIV and found that youth displayed complex perceptions of body satisfaction. Notably, 41% of overweight youth were satisfied with their body size, whereas 47% of normal-weight youth desired a larger body size, highlighting age-specific patterns in body dissatisfaction. Conversely, studies focusing on older adults with HIV have reported heightened concerns related to weight gain, facial lipoatrophy, and visible signs of aging. Wu et al. [33] demonstrated that facial lipoatrophy was a significant factor impacting self-perceived body image, mental, and physical health (*r* = 0.33 with MOS-HIV (Medical Outcomes Study HIV) and *r *= 0.26 with physical domains), reinforcing the psychosocial burden of ART-induced changes in body composition among older individuals.

Psychiatric conditions, including depression, anxiety, and Body Dysmorphic Disorder (BDD), frequently co-occur with HIV and contribute to heightened body dissatisfaction. A study by Lamb et al. [28] showed that 95% of participants had at least one psychiatric diagnosis, and 73% had two or more diagnoses, with major depressive disorder (46%) and generalized anxiety disorder (34%) as the most common conditions. Raggio et al. [39] showed that 70% of participants met the clinical cutoff for depression (CES-D ≥ 16; M = 20.5, SD = 1.3), and 43% met the higher cutoff (≥ 22). Additionally, 11% met the clinical cutoff for anxiety (STAI-S ≥ 55; M = 37.2, SD = 11.7). Similarly, Klimek et al. [26] reported that 95.5% (*N*= 42) of their participants met criteria for at least one psychiatric diagnosis, assessed by the Structured Clinical Interview for the Diagnostic and Statistical Manual of Mental Disorders (DSM-IVTR). Blashill et al. [28] documented high incidences of psychiatric conditions among PLWH, as 72.7% of participants had more than one DSM-IV diagnosis, including body dysmorphic disorder (68.2%), Major depressive disorder (45.5%), generalized anxiety disorder (34.1%), and dysthymic disorder (27.3**%**). The intersection of stigma, social exclusion, and depressive symptoms further exacerbates negative body image perceptions, with individuals experiencing both stigma and depression being more than twice as likely to report severe body dissatisfaction compared to those without these psychological stressors [29]​. Several studies indicated an association between internalized stigma and indices of poor body image (e.g., Brewster et al. [49] and Hart et al. [50]). Studies such as Palmer et al. [29] reported strong associations between HIV-related stigma and body image concerns, particularly among individuals who experience depressive symptoms, as the probability of positive body image was 68% when there was no stigma or depression. Fingeret et al. [27] highlighted that the stigma surrounding physical changes due to HIV treatment often led to social withdrawal and heightened self-consciousness about appearance.

On the other hand, body image may influence treatment adherence, mental health, and health-related quality of life among PLWH. Luzi et al. [45] reported that female sexual dysfunction (FSD) was directly linked to body image dissatisfaction​. Similarly, among HIV-positive gay and bisexual men, body dissatisfaction has been associated with increased methamphetamine use, suggesting a pattern of self-medication to cope with body image distress [41]​. Nutritional concerns further complicate these challenges; Wilkins et al. [44] found that while many HIV-positive youth classified as overweight were content with their body size, those with normal BMIs often desired a larger body, underscoring the unique psychosocial dimensions of body image in this population​. Lastly, among men diagnosed with AIDS, greater body dissatisfaction predicted higher depressive symptoms (B = − 7.30, t = − 6.10, *p* < 0.00001), which in turn predicted lower adherence to HAART (B = − 0.10, t = − 2.52, *p*= 0.01) [20].

### Body Image Interventions

A key approach that stands out across a few studies was the use of Cognitive Behavioral Therapy for Body Image and Self-Care (CBT-BISC), a structured intervention designed to simultaneously address body image concerns and self-care behaviors, including ART adherence. In Blashill et al.‘s (2017) study, all participants underwent Session 1 of Life-Steps, a single-session intervention that focuses on ART adherence and defining sexual health goals. Then, participants were assigned to either the Enhanced Treatment as Usual (ETAU) or CBT-BISC condition. In the ETAU condition, participants engaged in biweekly meetings with the project coordinator over the following three months. These sessions, lasting approximately 15 minutes, involved reviewing ART adherence via Wisepill data, addressing errors, and providing brief mental health referrals. Participants in the CBT-BISC condition received weekly individual sessions over three months (12 total sessions), with each session lasting around 50 minutes. CBT-BISC followed a structured manual, incorporating seven modules that targeted both body image and ART adherence. The first session was dedicated to orienting participants to the CBT framework and body image concepts. Subsequent modules integrated a range of cognitive behavioral techniques, such as mindfulness, perceptual retraining, cognitive restructuring, and exposure-based interventions to reduce body image disturbance. These strategies aimed to address negative body image and improve the participants’ overall well-being, which could, in turn, positively influence ART adherence. Sessions also included a review of adherence data, with body image discussions being framed around participants’ goals for ART adherence. The final module, relapse prevention, focused on reviewing learned skills and preparing participants for potential future challenges [31].

Blashill et al. [31] further reinforced the effectiveness of CBT-BISC, showing that the intervention led to substantial reductions in body image disturbance in terms of squared Difference in Pre-Post Change Scores (dppc2) (3 months: b = − 0.88, SE = 0.25, *p* < 0.00, dppc^2^ = 1.34; 6 months: b = − 1.0, SE = 0.25, *p* < 0.001, dppc^2^ = 1.52), sustained ART adherence improvements (b = 8.8, SE = 3.3, 95% CI [2.0, 15.6], *p* = 0.01; dppc^2^ = 0.94), and significant decreases in depressive symptoms (3 months: b = − 4.9, SE = 2.8, 95% CI [− 10.6, 0.7], *p* = 0.086; dppc^2^ = 0.87; 6 months: b = − 7.7, SE = 2.9, 95% CI [− 13.3, − 2.1], *p* = 0.008; dppc^2^ = 1.17). In another study, Participants in the CBT-BISC group, which specifically targeted body image distress, demonstrated significantly higher on-time adherence to ART (b = 8.8, SE = 3.3, 95% CI [2.0, 15.6], *p* = 0.01, dppc^2^= 0.94) compared to those in the control group. This disparity may contribute to disease progression and a declining self-perception of health [32].

In the Lamb et al. [30] study, participants in the CBT-BISC group showed statistically significant reductions in body image disturbance, which were sustained at both three-month and six-month follow-ups. In this study, CBT-BISC scores decreased from 25.36 (SD = 6.19) at baseline to 8.24 (SD = 8.95) at three months with Cohen’s d of 2.39, and 8.08 (SD = 10.08) at six months with Cohen’s d of 2.09. Also, participants assigned to CBT-BISC reported greater reductions in body-image disturbance from baseline to immediate follow-up compared to those in ETAU (b = − 14.13, SE = 2.43, t = − 5.79, *p* < 0.0001). Notably, these reductions in body image concerns positively influenced ART adherence (b = − 1.41, SE = 0.24, t = − 2.35, *p* = 0.019), highlighting a crucial link between body image and HIV treatment engagement. At six months post-treatment, 91% of participants in the CBT-BISC group maintained their improvements, compared to only 27% in the control group receiving ETAU. Additionally, on-time ART adherence significantly improved in terms of squared Difference in Pre-Post Change Scores (dppc^2^) (dppc^2^ = 0.94, *p* = 0.01), along with reductions in depressive symptoms (dppc^2^ = 1.17, *p* = 0.008) and increased global functioning (dppc^2^ = 3.39, *p* < 0.001).f

Moreover, Klimek et al. [28] explored the specific skills-based mechanisms driving CBT-BISC efficacy, demonstrating that reductions in avoidance and appearance-fixing behaviors were significant contributors to body image improvement. However, their findings emphasized that acceptance and cognitive reappraisal strategies were the strongest mediators of long-term success (β = −0.47, *p* = 0.001), accounting for the most variance in body image improvements across conditions. This suggests that CBT-BISC is most effective when it enhances adaptive cognitive processing and reduces maladaptive body image-related coping strategies.

## Discussion

This scoping review explored body image concerns and associated factors among PLWH. By analyzing 26 studies conducted between 2004 and 2024, key themes were identified across measurement tools, sociodemographic characteristics, biopsychosocial factors and consequences, and intervention strategies. The findings underscore the multifaceted nature of body image issues in PLWH and reveal significant gaps in the literature that warrant further investigation.

### Key Findings

As shown in Table 4, the findings revealed a high prevalence of body image dissatisfaction and disturbances among PLWH, such as a study conducted by C. Martins et al. [12]that reported rates of body dissatisfaction as high as 74.3%. Body image was associated with physical and clinical factors such as ART adherence and lipodystrophy [25, 27, 29, 31, 35, 36, 39, 40, 45, 46], psychological factors such as anxiety, depression, and stress [5, 26–29, 40], and sociological factors, including stigma and social support [36, 40, 42, 45].

**Table 4:** Statistical reports of included studies

ID	Citation	Statistical Method	Statistics Reported
1	Blashill & Vander Wal [35]	One-way analysis of variance (ANOVA)	Significant main effect for HIV/AIDS status on depressed mood, F(2, 251) = 4.4, *p* < 0.05. Significant main effect for age, F(2, 251) = 26.9, *p* < 0.0001. Follow-up Univariate ANOVAs identified which specific MBSRQ subscales differed significantly: Illness Orientation: F(2, 248) = 11.2, *p* < 0.0001, η² = 0.08, Fitness/Health Evaluation: F(2, 248) = 4.2, *p* < 0.05, η² = 0.03. No significant differences for: Appearance Evaluation (F = 2.0), Appearance Orientation (F = 0.13), Fitness/Health Orientation (F = 0.49).
2	Lima et al. [37]	Independent t-test/Mann–Whitney U Test. Analysis of covariance (ANCOVA). Multiple linear regression analysis.	Males with HIV showed lower weight (42.2 ± 11.7 vs. 52.1 ± 12.3 kg), height (150.6 ± 12.1 vs. 161.6 ± 8.2 cm), subscapular (7.8 ± 4.4 vs. 10.4 ± 6.1 mm), abdominal (11.1 ± 8.5 vs. 17.9 ± 7.9 mm), arm circumference (21.9 ± 3.4 vs. 24.2 ± 3.9 cm), and Total Estimated Subcutaneous Reserve (TESR) (0.90 ± 0.25 vs. 1.05 ± 0.25); all *p* < 0.05. Adolescents wishing to increase body size had lower BMI (17.5 ± 0.5), Σ4 skinfolds (41.7 ± 4.3 mm), Upper Arm Muscle Area (UAMA) (40.4 ± 2.0 cm²), compared to those wishing to reduce size (BMI = 22.6 ± 0.9, Σ4 = 78.4 ± 7.5 mm, UAMA = 55.1 ± 3.3 cm²); *p* < 0.001 across indicators. Key predictors of Body Image were: gender (β = − 0.52), age (β = 0.18), weight (β = 0.07), BMI (β = − 0.19), UAMA (β = − 0.08), and subscapular skinfold (β = − 0.06); the strongest standardized effects were weight (βstd = 0.70) and UAMA (βstd = − 0.54).
3	Fingeret et al. [27]	Logistic Regression and Correlations	Body image concerns were positively correlated with: Depression (*r* = 0.41, *p* < 0.001), Anxiety (*r* = 0.33, *p* = 0.003), Perceived stress (*r* = 0.26, *p* = 0.02), and negatively correlated with social support (*r* = − 0.35, *p* = 0.002). Logistic regression found Moderate vs. high/low body image concerns(OR = 3.54, 95% CI = 1.12–11.21, *p* = 0.03 (unadjusted)). After adjusting for depression, anxiety, social support, stress, and treatment group (OR = 7.13, 95% CI = 1.54–33.15 (still significant)).
4	Junior et al. [38]	Cramér’s V test and Analysis of covariance (ANCOVA)	Among males, 40% were satisfied, 23.3% wanted to reduce weight, and 36.7% wanted to increase weight; among females, 40% were satisfied, 28.6% wanted to reduce, and 31.4% wanted to increase weight. There was no significant sex difference in body image categories (Cramér’s V = 0.579, *p* = 0.861). ANCOVA showed significant group differences in girls’ body image categories (*p* < 0.001) but not in boys. Effect sizes were moderate to large (η² = 0.275–0.344) with strong model fit (Adjusted R² = 0.503–0.613). The association between sex and body image was weak and nonsignificant (Cramér’s V = 0.579, *p* = 0.861). Significance was set at *p* ≤ 0.05, and Cohen’s d was noted but not detailed for body image comparisons. Overall, body image dissatisfaction was statistically linked to higher fat mass in girls only.
5	Corless et al. [34]	Analysis of Variance (ANOVA) and Pearson Correlation Coefficients (r)	Body image and health transition (women): *r* = 0.52, *p* = 0.02, Body image and overall health (men): *r* = 0.49, *p* = 0.01, Body image and mental health (men): *r* = 0.57, *p* = 0.003. Analysis of Variance (ANOVA) was used to test gender and disease-status differences in body image scores. Women had significantly higher body image scores than men (F(1, 37) = 5.41, *p* = 0.03). No significant difference between HIV-positive and AIDS participants (F(1, 37) = 1.56, *p* = 0.22).
6	Lamb et al. [30]	Latent Difference Score (LDS) Mediation Modelling	Participants in the CBT-BISC condition had significantly greater reductions in body image disturbance (b = − 14.13, SE = 2.43, t = − 5.79, *p* < 0.0001). Reductions in body image disturbance predicted improvements in ART adherence(b = − 1.41, SE = 0.24, t = − 2.35, *p* = 0.019). The indirect effect of treatment on adherence through reductions in body image disturbance was statistically significant (b = 20.01, SE = 9.11, t = 2.19, *p* = 0.028), indicating that body image disturbance functioned as a mediator of the treatment’s effect on adherence.
7	Gholizadeh et al. [42]	Generalized linear model (GLM)	The overall model was significant (χ²(3) = 80.36, *p* < 0.001), showing that body image factors together influenced condomless anal sex. Body dissatisfaction alone (b = 0.02, *p* = 0.46) and appearance investment alone (b = −0.07, *p* = 0.78) did not significantly affect the behaviour (condomless anal sex). interaction (b = 0.08, *p* = 0.033) showed that when appearance investment was high, greater body dissatisfaction led to more condomless anal sex, but when appearance investment was low, it led to fewer acts.
8	Blashill & Vander Wal [20]	Mediation and Moderated Mediation Models, and Pearson Correlations	Greater body dissatisfaction is strongly associated with higher depression (*r*= − 0.482, *p*< 0.001).Body dissatisfaction and HAART non-adherence: *r* = 0.122, *p* = 0.177. Body image dissatisfaction predicted higher depression (B = − 7.30, t = − 6.10, *p* < 0.00001), but depression did not significantly mediate HAART non-adherence overall (bootstrapped 95% CI = − 0.05 to 0.38). For men diagnosed with AIDS, depression mediated the effect of body dissatisfaction on non-adherence (B interaction = 0.05, t = 2.2, *p* = 0.03; 95% CI = 0.06 to 0.75), but not for those without AIDS (95% CI = − 0.23 to 0.18).
9	C. Martins et al. [12]	Multivariate analysis conducted using Poisson Regression,	The analysis revealed that a negative self-perception of body image (NSPBI) was significantly associated with low weight (PR = 3.84; CI: 1.68–8.82), obesity (PR = 2.92; CI: 1.26–6.75), female gender (PR = 2.3; CI: 1.4–3.8), and the presence of depressive symptoms (PR = 2.88; CI: 1.59–5.20). Body changes in specific areas such as the legs, abdomen, and face were also significantly associated with NSPBI (*p* < 0.05).
10	Raggio et al. [5]	One-Way Analysis of Variance (ANOVA)	More severe/diffuse lipodystrophy was associated with: Greater body image disturbance(F(2,54) = 11.86, *p* < 0.001, partial η² = 0.313), Higher anxiety levels (F(2,52) = 3.82, *p* = 0.029, partial η² = 0.133), and A trend toward higher depression (F(2,55) = 2.58, *p* = 0.085). No significant association with social support (F(2,55) = 1.44, *p* = 0.246).
11	Klimek et al. [28]	Latent Difference Score Mediation Analysis (LDS mediation)	CBT-BISC significantly reduced body image disturbance compared to E-TAU (β = −0.24, *p* = 0.036). CBT-BISC participants also showed: Reduced avoidance (β = −0.34, *p* = 0.005), Reduced appearance fixing (β = −0.24, *p* = 0.049), and Improved acceptance and cognitive reappraisal (β = 0.32, *p* = 0.01). However, only acceptance and cognitive reappraisal significantly predicted improvements in body image disturbance (β = −0.47, *p* = 0.001). The indirect (mediated) effect through acceptance and cognitive reappraisal was statistically significant (β = −0.15, *p* = 0.03), while effects through avoidance and appearance fixing were not. Effect sizes showed that the acceptance and reappraisal pathway (ES = − 0.607) was 1.5–1.6 times stronger than the behavioural pathways.
12	Blashill et al. [31]	Generalized Linear Modelling (GENLIN)	CBT-BISC participants showed a large drop in body image disturbance vs. ETAU (3 mo b = − 13.6, *p* < 0.001; 6 mo b = − 11.9, *p* < 0.001). Depression declined significantly by 6 months (b = − 7.7, *p* = 0.008). ART adherence improved during treatment (b = 8.8, *p* = 0.01). Global functioning rose markedly (3 mo b = 12.3, *p* < 0.001; 6 mo b = 15.5, *p* < 0.001).
13	Blashill et al. [40]	Structural equation modelling (SEM)	Lipodystrophy and appearance investment were associated with elevated body image disturbance (B = 0.12, SE = 0.001, 95% CI: 0.04–0.20; B = 0.42, SE = 0.006, 95% CI: 0.16–0.69). Body image disturbance was associated with increased depressive symptoms (B = 0.45, SE = 0.003, 95% CI: 0.32–0.57) and lowered condom use self-efficacy (B = − 0.37, SE = 0.005, 95% CI: −0.60 to − 0.14). Depressive symptoms were associated with poorer ART adherence (B = − 0.57, SE = 0.004, 95% CI: −0.90 to − 0.29). Lower condom use self-efficacy was associated with increased HIV sexual risk behaviors (B = − 1.9, SE = 0.023, 95% CI: −3.1 to − 0.58).
14	Zanlorenci et al. [36]	t-tests, ANOVA, Pearson and Spearman correlations, and multiple linear regression	Body image dissatisfaction increased with younger age (*r* = − 0.462; *p* = 0.013). Females with less than 2 hours of screen time on weekends had a larger current silhouette (*t*(30) = 2.56, *p* = 0.015). Overweight females had a larger ideal silhouette compared to eutrophic (normal weight) ones (F (2, 29) = 5.48, *p* = 0.007). No significant differences for males in these comparisons. Lower subscapular skinfold (β = −0.236; 95% CI − 0.457 to − 0.016; *p* = 0.036) , Higher calf skinfold (β = 0.231; 95% CI 0.001 to 0.462; *p* = 0.049) , Pre-pubertal maturation (β = −1.595; *p* < 0.01), Higher economic level (β = −1.671; *p* < 0.01), Lower CD4 + lymphocytes (β = −0.119; *p* < 0.01), Lower viral load (β = −1.489; *p* < 0.01), Less physical activity (β = 0.824; *p* < 0.05), and more screen time (computer/video games) (β = 2.023–4.037; *p* < 0.01)were independently associated with greater body image dissatisfaction.
15	Huang et al. [39]	Wilcoxon rank-sum tests and ANOVA. Stepwise multiple linear regression	HIV-infected men with lipodystrophy had significantly poorer body image (BIQLI = − 0.3 ± 0.2) than those without (BIQLI = + 0.6 ± 0.2, *p* < 0.0001). They also showed greater bodyimage distress (SIBID-S = 2.1 ± 0.1 vs. 1.4 ± 0.1, *p* < 0.0001). Facial fat atrophy caused higher bodyimage distress (SIBID-S = 2.0 ± 0.1, *p* = 0.003). Neck and abdominal fat changes also worsened image (SIBID-S = 2.4 ± 0.3, *p* = 0.02; BIQLI = − 0.2 ± 0.2, *p* = 0.004). Buttock and breast fat hypertrophy affected Body Image Quality of Life Inventory (BIQLI = + 0.9 ± 0.6, *p* = 0.02; −0.2 ± 0.3, *p* = 0.03). Clinical lipodystrophy predicted poorer body image (β = −0.33, *p* = 0.02), as did being Caucasian (β = −0.33, *p* = 0.005) and having depression/anxiety (β = −0.38, *p* = 0.007). Age had a small, nonsignificant positive effect (β = +0.19, *p* = 0.18). Perceived body changes (β = −0.23, *p* = 0.10) and health rating (β = −0.01, *p* = 0.16) were not significant predictors.
16	Guaraldi et al. [46]	Correlations and t-test	Body satisfaction (Q7) negatively correlated with mental health (*r* = − 0.34, *p* < 0.0001), vitality (*r* = − 0.29, *p* < 0.0001), and overall QoL (*r* = − 0.33, *p* < 0.0001). Body satisfaction (ABCD Q7) was lower in LD+ (3.78 ± 1.06) vs. LD− (2.65 ± 0.70; *p* < 0.001). Total psychosocial distress (Q8 summary) was worse in LD+ (67 ± 19) vs. LD− (85 ± 15; *p* < 0.0001). Body image satisfaction was significantly correlated with several HRQOL dimensions: Pain: positively correlated (*r* = 0.16, *p* = 0.012). Physical function: negatively correlated (*r* = − 0.20, *p* = 0.0013). Role function: negatively correlated (*r* = − 0.17, *p* = 0.0052). Social function: negatively correlated (*r* = − 0.27, *p* < 0.0001). Mental health: negatively correlated (*r* = − 0.34, *p* < 0.0001). Vitality: negatively correlated (*r* = − 0.29, *p* < 0.0001). Health distress: negatively correlated (*r* = − 0.38, *p* < 0.0001). Cognitive function: negatively correlated (*r* = − 0.27, *p* < 0.0001). Overall quality of life: negatively correlated (*r* = − 0.33, *p* < 0.0001). Physical health summary score: negatively correlated (*r* = − 0.25, *p* = 0.0003). Mental health summary score: negatively correlated (*r* = − 0.39, *p* = 0.0001). Non-significant associations were observed for quality of life (*r* = 0.10, *p* = 0.092), BMI (*r* = − 0.084, *p* = 0.259), and CD4 + count (*r* = − 0.038, *p* = 0.600).
17	Luzi et al. [45]	ANOVA and Multivariate Linear Regression	Severity of self-perceived central fat accumulation increased with FSD severity (F = 3.47, *P* = 0.02), indicating a body image effect. Lubrication (vaginal moisture) was negatively associated with body image dissatisfaction (β = − 0.49, 95% CI − 0.88 to − 0.10, *P* < 0.05). Orgasm was negatively associated with body image dissatisfaction (β = − 0.58, 95% CI − 1.00 to − 0.16, *P* < 0.01).
18	M. Martins et al. [11]	Pearson correlation, Independent-samples t-tests, and ANOVA	Physical pain related to appearance correlated positively with DAS-24 total score (*r* = 0.61, *p* < 0.05). Physical limitation related to appearance correlated similarly (*r* = 0.68, *p* < 0.05). Both findings indicate that higher reported pain or limitation corresponded with greater appearance-related distress. A Pearson correlation showed no association between age and DAS-24 distress score (*r* = − 0.001, *p* = 0.98). Women (M = 37.42 ± 16.89) scored higher than men in DAS-24 (M = 26.77 ± 14.84), *t*(397) = − 6.27, *p* < 0.001, *d* = 0.67. Participants who identified a bothersome physical feature (M = 40.43 ± 14.54) scored higher than those who did not in DAS-24 (M = 19.96 ± 10.61), *t*(398) = 15.98, *p* < 0.001, *d* = 1.61. One-way ANOVAs assessed DAS-24 scores by body site and lipodystrophy category showed no significant differences were found by body region affected (F(5, 201) = 0.32, *p* = 0.90). Also, no significant differences by lipodystrophy type (F(6, 194) = 0.44, *p* = 0.85) or other appearance features (F(6, 89) = 0.95, *p* = 0.46).
19	Palmer et al. S. [29]	Chi-square χ² and Wilcoxon tests and Multivariate Logistic Regression.	Negative body image was reported by 47% of participants. ART adherence ≥ 95% was significantly higher among participants with positive body image (66.7% vs. 45.9%, χ² = 14.33, *p* < 0.001). Positive body image among HIV-positive adults on ART was significantly associated with male gender (χ² = 11.64, *p* < 0.001), higher education (χ² = 13.95, *p* < 0.001), stable housing (χ² = 6.36, *p* = 0.012), food security (χ² = 15.83, *p* < 0.001), and having a regular partner (χ² = 5.27, *p* = 0.022). High stigma with depression was associated with a lower likelihood of positive body image (multivariate logistic regression, AOR = 0.42, 95% CI [0.21–0.81], *p* = 0.009). Higher sexual function scores were strongly associated with positive body image (Wilcoxon rank-sum test, median 67 [IQR 50–75] vs. 50 [33–58], Z = − 8.91, *p* < 0.001).
20	Sackey et al. [25]	One-way ANOVA	Body image did not differ by fruit availability (F(2, 37) = 0.38, *p* > 0.05), vegetable availability (F(2, 37) = 0.69, *p* > 0.05), soft drink availability (F(4, 35) = 0.69, *p* > 0.05), and overall diet quality (F(2, 36) = 0.80, *p* > 0.05).
21	Sharma et al. [26]	Chi-square test and Logistic Regression	Negative body image prevalence differed by BMI category: lean/normal = 24%, overweight = 30%, obese = 49% (χ² = 16.9, *p* ≤ 0.001); no difference by HIV status (χ² = 0.42, *p* > 0.05). BMI (kg/m²) was positively associated with negative body image (adjusted OR = 1.05, 95% CI 1.01–1.09, *p* < 0.05). Self-rated fair/poor health increased odds of negative body image compared with excellent/good health (adjusted OR = 2.07, 95% CI 1.34–3.20, *p* < 0.01). Depression (CES-D ≥ 16 = depressive symptoms) was associated with negative body image (adjusted OR = 1.64, 95% CI 1.08–2.48, *p* < 0.05). Erectile dysfunction (difficulty achieving/maintaining erection in prior two weeks) was associated with negative body image (adjusted OR = 1.85, 95% CI 1.23–2.80, *p* < 0.01). HIV status was not significantly associated with negative body image (OR = 1.15, 95% CI 0.79–1.66, *p* > 0.05).
22	Martinez et al. [32]	Paired t-test and One-way ANOVA.	Current BIS scores were higher (worse body image) than pre-HIV (33.2 ± 10.8 vs. 22.3 ± 10.8; paired t = 9.0, *p* < 0.001). Current self-perception was more favourable than perceived societal view (33.2 ± 10.8 vs. 42.6 ± 10.5; paired t = − 9.14, *p* < 0.001). Presence of symptomatic HIV was associated with higher BIS scores (poorer body image; t = − 5.0, *p* < 0.001). CD4 count (continuous or categorical) and plasma HIV RNA (log₁₀ copies/mL) were not significantly associated with BIS score (*p* > 0.05). Age, gender, ethnicity, HIV risk factor, partnership status, and duration of infection were not significantly related to BIS score.
23	Theodore et al. [41]	Pearson Correlations and Regression Analyses	Methamphetamine Use × Body Dissatisfaction: *r* = 0.41, *p* < 0.01. No significant associations with age, ethnic group, education, income, HIV viral load, or CD4 count. In multivariate regression, body dissatisfaction remained a significant predictor of Methamphetamine use (β = 0.244, *p* < 0.05), explaining an additional 5.4% of variance.
24	Wilkins et al. [44]	Chi-square tests	Body Image Discrepancy (BID) by HIV route showed that 31.9% of behaviorally infected youth desired a larger body and 32.8% a smaller one, whereas 20.8% of perinatally infected youth desired a larger and 50.0% a smaller body. MSM (men who have sex with men) preferred larger body size (40.2%) more often than smaller (22.0%) or current size (37.8%), similar to non-MSM patterns
25	Wu et al. [33]	t-tests and correlation	Participants with lower mental health scores (< 45.2) had lower FAI scores (20.0) than those with higher scores (> 45.2: 29.7; *p* < 0.01). Participants with lower physical health scores (< 44.0) had lower FAI scores (21.2) than those with higher scores (> 44.0: 28.5; *p* < 0.05). Perceived facial appearance was positively associated with both mental health (*r* = 0.33, *p* < 0.01) and physical health (*r* = 0.26, *p* < 0.05), with a stronger relationship observed for mental health.
26	Soares et al. [43]	Spearman Correlation and Mann–Whitney U test	Lower body image scores were associated with loss of facial fat (median = 50.0 [40.2–60.0] vs. 70.0 [70.0–70.0], *p* = 0.004), loss of arm and thigh fat (arms *p* = 0.006; thighs *p* < 0.001), buttock fat loss/sagging and larger waistlines (*p* < 0.001 and *p* = 0.001), and visible veins and lipomas (*p* < 0.001 and *p* = 0.046). Triceps skinfold thickness correlated positively with body image (ρ = 0.14, *p* = 0.031).

**Fig. 2:**
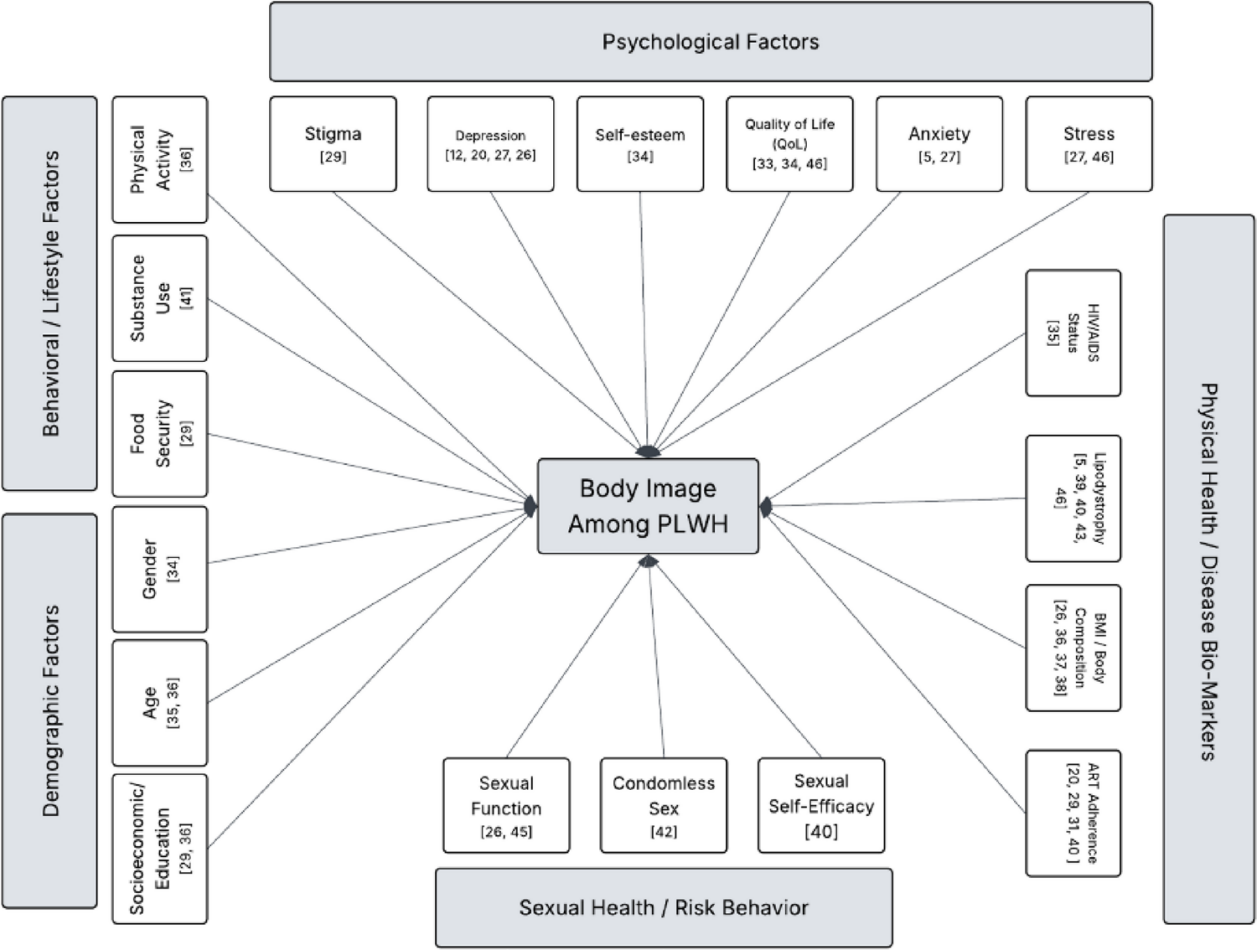
Thematic structure of the review findings, including multifaceted determinants of Body Image among PLWH

As demonstrated in Fig. 2, body image disturbance was a widespread concern among PLWH, primarily driven by lipodystrophy, especially changes in facial and abdominal fat. These changes were consistently linked to lower body satisfaction, increased distress, and diminished quality of life across various studies. Physical changes, such as lipodystrophy and altered body composition, were prominent risk factors for developing negative body image among PLWH. These findings likely arise because lipodystrophy, a visible and stigmatized side effect of ART, directly affects appearance, leading to heightened self-awareness and dissatisfaction. BMI also emerged as a critical factor associated with body image. Studies like those by Junior et al. [38] noted that both lower and higher BMI values impacted body satisfaction, though through different mechanisms. Lower BMI was associated with perceptions of frailty and vulnerability to illness, while higher BMI raised concerns about stigma and societal ideals of body aesthetics.

Additionally, depression and anxiety were closely related, exhibiting moderate-to-strong positive correlations that indicate a bidirectional influence. Importantly, negative body image independently contributed to non-adherence to ART, diminished sexual self-efficacy, and higher-risk behaviors for HIV transmission, particularly when combined with a high investment in appearance.

Sociodemographic characteristics, including gender, age, and racial composition, appeared to influence body image perceptions. Women and younger individuals reported more significant dissatisfaction, often exacerbated by lower socioeconomic resources, reduced physical activity, and increased screen time. The predominance of male participants in the reviewed studies reflects a gender bias in the existing literature. However, studies that included substantial female representation (e.g., Martins, C et al., [12]) reported that women with HIV exhibited higher rates of body dissatisfaction (81.2%) compared to men (65.6%). This discrepancy can be attributed to societal beauty standards that disproportionately emphasize physical appearance for women, amplifying the impact of HIV-related changes. This can also be explained by the negative effects of the mass media on body image perception, as body image dissatisfaction is strongly related to standards imposed by society and culture [51].

Furthermore, variations in racial and ethnic representation across studies suggest that cultural and contextual factors may play a role in shaping body image experiences, though this area remains underexplored. Junior et al. [38] noted that cultural norms surrounding body appearance and health perceptions could modulate body image concerns, particularly in settings with strong societal expectations around physical appearance. Such cultural pressures may exacerbate or mitigate body image concerns depending on the societal context and support structures available [52].

In contrast, factors such as social support, food security, and stable housing acted as protective elements. Notably, cognitive-behavioral interventions such as CBT-BISC have shown substantial, lasting improvements in body image, primarily through enhanced acceptance and cognitive reappraisal, subsequently benefiting medication adherence, mental health, and overall functioning. Lamb et al. [30] reported that CBT-BISC not only improved body image perceptions but also enhanced positive coping mechanisms and adaptive emotion regulation strategies such as cognitive reappraisal and acceptance. Positive self-image and resilience were further bolstered by participation in structured interventions and access to affirming healthcare environments [30]. This suggests that supportive interventions can empower PLWH to challenge negative self-perceptions and build resilience against external judgments. Furthermore, social networks and community support emerged as critical buffers against the adverse effects of stigma and physical changes.

Together, these findings highlighted body image as a critical, modifiable factor influencing both psychological well-being and clinical outcomes in HIV care. Conversely, biomarkers of HIV disease, such as CD4 count and viral load, showed no relationship with body image, regardless of treatment-related changes. Additionally, dietary quality, availability of fruits and vegetables, and access to soft drinks did not appear to have an impact. Ethnicity, HIV risk factors, and duration of infection were also found to be non-significant in many analyses. Finally, social support did not moderate the relationship between lipodystrophy and body image disturbance in some samples. These null findings emphasize that perceived changes rather than objective assessments, as well as psychological factors rather than purely clinical or demographic variables, are the primary drivers of body image concerns among PLWH.

In conclusion, the reviewed studies suggest that addressing both risk and protective factors through targeted interventions could significantly mitigate body image concerns and improve overall well-being in PLWH.

### Knowledge Gaps

The majority of included studies were conducted in a limited number of countries, predominantly the United States, Brazil, Canada, and Italy, which limits the generalizability of findings to other regions, particularly low-income countries and non-Western settings. Our findings also showed that gender differences in body image dissatisfaction were often inadequately examined, with female participants experiencing higher levels of distress but receiving less research attention. In this regard, investigating how cultural and gender norms shape body image perceptions could lead to more targeted interventions for at-risk groups.

In seven studies, key confounders were not adequately measured or controlled. Factors such as HIV disease progression, medication side effects, mental health conditions (e.g., depression, anxiety), and socio-cultural influences on body image were often overlooked or inadequately adjusted in statistical analyses. Studies that did not stratify analyses to account for these confounders may have reported spurious associations between body image concerns and other psychological or health-related variables. For instance, studies that explored gender differences in body image dissatisfaction among PLWH often did not control for differential effects of ART-related side effects in men and women, leading to overgeneralized conclusions about gender-based disparities. Similarly, studies examining self-esteem and body dissatisfaction frequently lacked adjustments for psychosocial stressors (e.g., stigma, social support), which could be primary contributors to negative body image rather than biological or medical factors alone.

Additionally, the predominance of cross-sectional designs limits the ability to establish causal relationships between body image and its associated factors. Longitudinal studies are urgently needed to establish causal relationships between body image concerns and factors such as ART adherence, mental health outcomes, and social determinants to examine how body dissatisfaction evolves over time in response to ART side effects, changes in disease progression, and psychosocial influences.

The review also identified a lack of standardized measurement tools for assessing body image among PLWH. Sixteen different instruments were used across the studies, including MBSRQ, ABCD, and the Silhouette Scale. While these tools provided valuable insights, inconsistencies in their application limited the comparability of findings. Many studies failed to report the psychometric properties of their instruments or used adapted versions without validation, raising concerns about measurement reliability. Additionally, the lack of culturally tailored tools further reduced the applicability of findings across diverse populations. The reliance on self-reported BMI in a substantial portion of studies also highlights a key methodological limitation in body image research among PLWH. Given the known biases associated with self-reporting, future studies should prioritize clinician-measured BMI or, where feasible, incorporate advanced body composition assessments, which would offer a more precise evaluation of body composition, allowing for a more nuanced understanding of how BMI, fat distribution, and body image interact in this population.

Finally, the interplay between stigma, mental health, and body image in PLWH requires further investigation. Several studies in our review demonstrated that HIV-related stigma exacerbates body dissatisfaction and psychological distress, yet few studies examined stigma-reduction strategies as a means of improving body image outcomes. Only two studies provided an integrated intervention to address body image among PLWH, which underscores the effectiveness of this strategy in improving mental and physical outcomes in this population. However, the study setting which were in a single country/cultural context limited the generalizability of the findings.

Future research should explore alternative intervention approaches, including digital health interventions, community-based support programs, and integrated mental health services within HIV care. Also, studies should assess structural and policy-level interventions aimed at reducing stigma in healthcare and community settings, as well as the potential benefits of peer-led and group-based support models for PLWH struggling with body image concerns. Addressing these research gaps will be essential for developing comprehensive, culturally sensitive interventions that enhance the well-being and treatment outcomes of PLWH across diverse contexts.

### Our Study Limitations

A key strength of this review lies in its comprehensive synthesis of studies spanning over two decades, utilizing diverse methodologies, and considering international findings. This is the first review to comprehensively address the multidimensional aspects of body image concerns in PLWH. However, several limitations should be acknowledged. The decision to limit our search to only four databases may have restricted the scope of the literature review and potentially excluded relevant studies indexed in other databases. Additionally, the inclusion of only English-language, peer-reviewed studies, along with the exclusion of grey literature, may have led to the omission of valuable insights that could have further enriched the findings. For future literature reviews, we recommend the expansion of the search to include a wider range of databases and sources, including grey literature and non-English publications, to capture a more diverse set of studies. Moreover, incorporating studies from underrepresented geographic regions and targeting more interventional studies could enhance the generalizability and depth of findings on body image among PLWH.

### Clinical and Public Health Implications

The findings of this review underscore the critical need to integrate body image assessments into routine HIV care to identify and address body image-related distress among PLWH. Given the strong associations between body dissatisfaction, mental health conditions (e.g., depression, anxiety, BDD), and ART adherence, a holistic, multidisciplinary approach is essential. Incorporating regular screening for body image concerns within HIV care settings can facilitate the early identification of at-risk individuals, allowing for timely psychological and medical interventions.

Intervention strategies such as CBT-BISC have demonstrated effectiveness in reducing body dissatisfaction and improving ART adherence, yet their accessibility remains limited. To maximize reach and impact, integrating low-resource, scalable interventions, including digital mental health programs, peer-led support groups, and community-based psychosocial interventions, may offer more sustainable solutions, particularly in resource-limited settings. Furthermore, given the gender and racial disparities in body image concerns, interventions must be culturally adapted and tailored to meet the specific needs of women, youth, and racial/ethnic minorities living with HIV, who often experience heightened body dissatisfaction and stigma.

From a public health perspective, addressing structural barriers such as stigma, discrimination, and socioeconomic inequalities is crucial for improving body image outcomes. Health policies that promote stigma reduction, such as public awareness campaigns, healthcare provider training, and inclusive messaging around HIV-related body changes, could help reduce the psychosocial burden associated with body dissatisfaction. Moreover, integrating mental health services into HIV care frameworks will be essential for ensuring that body image interventions are not only available but also widely accessible and sustainable. By addressing body image concerns through a comprehensive, patient-centered approach, healthcare systems can enhance mental health outcomes, improve ART adherence, and ultimately contribute to better overall well-being for PLWH.

## Conclusion

Body image concerns among PLWH are a complex issue shaped by a combination of biopsychosocial factors. This review identified key risk factors, including HIV-related bodily changes (e.g., lipodystrophy, BMI fluctuations, ART side effects), psychological distress (e.g., depression, anxiety, BDD), and social determinants (e.g., stigma). These concerns, in turn, influence ART adherence, mental health, and overall well-being. Protective factors such as strong social networks and targeted interventions like CBT-BISC have demonstrated potential in alleviating body image distress and improving self-perception among PLWH. Future research should focus on longitudinal studies, the development of validated body image measures for PLWH, and integrated healthcare approaches that incorporate both mental and physical health support. Addressing these research and clinical gaps will be essential for enhancing the well-being and quality of life of PLWH.

## Supplementary Information

Below is the link to the electronic supplementary material.

Supplementary Material 1
