# Supplementary material for: Body Image and Its Associated Factors among People Living with HIV: A Scoping Review and Implications for Integrated Care
Source: AIDS Behav. 2025 Dec 23;30(5):1610–34. doi: 10.1007/s10461-025-04991-6 (PMC13167905; doi:10.1007/s10461-025-04991-6)
Supplement: Supplementary file 1 — Supplementary Material 1 [file 10461_2025_4991_MOESM1_ESM.docx]

**Body Image and Its Associated Factors among People Living with HIV:**

**A Scoping Review and implications for integrated care**

**Atena Pasha^a,b^, Mohammad Jahanaray^c^, Xiaoming Li^a,b^, Shan Qiao^a,b^**

a. Department of Health Promotion, Education and Behavior, Arnold School of Public Health, University of South Carolina, Columbia, SC, USA.

b. South Carolina SmartState Center for Healthcare Quality, Arnold School of Public Health, University of South Carolina, Columbia, SC, USA.

c. School of Education, Virginia Commonwealth University, Richmond, VA, USA.

**Corresponding author:** Atena Pasha, Ph.D.

Department of Health Promotion, Education and Behavior,

University of South Carolina, Arnold School of Public Health,

915 Greene St, Room 529

Phone: 1-803-470-9181 E-mail: [atena.pasha@tamuk.edu](mailto:atena.pasha@tamuk.edu) ORCID: 0000-0001-5603-4961

**Supplementary Table 1a.** *Search strategy and keywords used for identifying studies related to people living with HIV and body image.*

| **Concept** | **Controlled Vocabulary** | **Keywords** |
| --- | --- | --- |
| PLWH | - "Acquired Immunodeficiency Syndrome"[Mesh] - "HIV"[Mesh] | - Persons living with AIDS - People living with AIDS - Persons living with HIV - People living with HIV - PLHIV - PLWH - PLWHA - Acquired immunodeficiency syndrome(s) - Acquired immune-deficiency syndrome(s) - Acquired immune deficiency syndrome(s) - AIDS - Human immunodeficiency virus(es) - HIV - HIV1 - HIV-1 - HIV2 - HIV-2 |
| Body Image | - “Body Image”[Mesh] - “Self-Concept”[Mesh:NoExp] | - Body image(s) - Body-image - Body integrity - Body schema(s) - Body representation(s) - Body dissatisfaction - Body satisfaction - Body esteem - Body self-esteem - Body appreciation - Body shame - Body preoccupation - Body discomfort - Body perception - Body insecurity - Body acceptance - Body confidence - Body concern - Body attitude - Body awareness - Body dysphoria - Body dysmorphia - Body dysmorphic - Body distortion - Body ideal - Self-image - Self-concept - Self-representation - Appearance evaluation - Appearance ~ (dis)satisfaction - Appearance anxiety - Appearance concern - Appearance-related anxiety - Appearance-related concern |

**Supplementary Table 1b.** *Search strategy and search string used in* ***PubMed****.*

| **Search #** | **Concept** | **Query** | **Results** |
| --- | --- | --- | --- |
| 1 | PLWH | "Acquired Immunodeficiency Syndrome"[Mesh] OR "HIV"[Mesh] OR “persons living with AIDS”[tiab] OR “people living with AIDS”[tiab] OR “persons living with HIV”[tiab] OR “people living with HIV”[tiab] OR PLHIV[tiab] OR PLWH[tiab] OR PLWHA[tiab] OR “acquired immunodeficiency syndrome*”[tiab] OR “acquired immune-deficiency syndrome*”[tiab] OR “acquired immune deficiency syndrome*”[tiab] OR AIDS[tiab] OR “human immunodeficiency virus*”[tiab] OR HIV[tiab] OR HIV1[tiab] OR HIV-1[tiab] OR HIV2[tiab] OR HIV-2[tiab] | 497,775 |
| 2 | Body Image | "Body Image"[Mesh] OR “body image*”[tiab] OR body-image[tiab] OR “body integrity”[tiab] OR “body schema*”[tiab] OR “body representation*”[tiab] OR “body dissatisfaction”[tiab] OR “body satisfaction”[tiab] OR “body esteem”[tiab] OR “body self-esteem”[tiab] OR “body appreciation”[tiab] OR “body shame”[tiab] OR “body preoccupation”[tiab] OR “body discomfort”[tiab] OR “body perception”[tiab] OR “body insecurity”[tiab] OR “body acceptance”[tiab] OR “body confidence”[tiab] OR “body concern*”[tiab] OR “body attitude*”[tiab] OR “body awareness”[tiab] OR “body dysphoria”[tiab] OR “body dysmorph*”[tiab] OR “body distortion”[tiab] OR “body ideal”[tiab] OR self-image[tiab] OR self-concept[tiab] OR self-representation[tiab] OR “appearance evaluation”[tiab] OR “appearance satisfaction”[tiab:~3] OR “appearance dissatisfaction”[tiab:~3] OR “appearance anxiety”[tiab] OR “appearance concern*”[tiab] OR “appearance-related anxiety”[tiab] OR “appearance-related concern*”[tiab] | 45,256 |
| 3 |  | #1 AND #2 | 533 |
| 4 | Language | #3 AND eng[la] | 511 |
| 5 | Date | #4 AND 2000/01/01:2024/12/31[pdat] | 456 |

("Acquired Immunodeficiency Syndrome"[Mesh] OR "HIV"[Mesh] OR "persons living with AIDS"[tiab] OR "people living with AIDS"[tiab] OR "persons living with HIV"[tiab] OR "people living with HIV"[tiab] OR PLHIV[tiab] OR PLWH[tiab] OR PLWHA[tiab] OR "acquired immunodeficiency syndrome*"[tiab] OR "acquired immune-deficiency syndrome*"[tiab] OR "acquired immune deficiency syndrome*"[tiab] OR AIDS[tiab] OR "human immunodeficiency virus*"[tiab] OR HIV[tiab] OR HIV1[tiab] OR HIV-1[tiab] OR HIV2[tiab] OR HIV-2[tiab]) AND ("Body Image"[Mesh] OR "body image*"[tiab] OR body-image[tiab] OR "body integrity"[tiab] OR "body schema*"[tiab] OR "body representation*"[tiab] OR "body dissatisfaction"[tiab] OR "body satisfaction"[tiab] OR "body esteem"[tiab] OR "body self-esteem"[tiab] OR "body appreciation"[tiab] OR "body shame"[tiab] OR "body preoccupation"[tiab] OR "body discomfort"[tiab] OR "body perception"[tiab] OR "body insecurity"[tiab] OR "body acceptance"[tiab] OR "body confidence"[tiab] OR "body concern*"[tiab] OR "body attitude*"[tiab] OR "body awareness"[tiab] OR "body dysphoria"[tiab] OR "body dysmorph*"[tiab] OR "body distortion"[tiab] OR "body ideal"[tiab] OR self-image[tiab] OR self-concept[tiab] OR self-representation[tiab] OR "appearance evaluation"[tiab] OR "appearance satisfaction"[tiab:~3] OR "appearance dissatisfaction"[tiab:~3] OR "appearance anxiety"[tiab] OR "appearance concern*"[tiab] OR "appearance-related anxiety"[tiab] OR "appearance-related concern*"[tiab]) AND eng[la] AND 2000/01/01:2024/12/31[pdat]

**Supplementary Table 1c.** *Search strategy and search string used in* ***PsycINFO****.*

| **Search #** | **Concept** | **Query** | **Results** |
| --- | --- | --- | --- |
| 1 | PLWH | ( DE "AIDS" OR DE "HIV" ) OR TI ( “persons living with AIDS” OR “people living with AIDS” OR “persons living with HIV” OR “people living with HIV” OR PLHIV OR PLWH OR PLWHA OR “acquired immunodeficiency syndrome*” OR “acquired immune-deficiency syndrome*” OR “acquired immune deficiency syndrome*” OR AIDS OR “human immunodeficiency virus*” OR HIV OR HIV1 OR HIV-1 OR HIV2 OR HIV-2 ) OR AB ( “persons living with AIDS” OR “people living with AIDS” OR “persons living with HIV” OR “people living with HIV” OR PLHIV OR PLWH OR PLWHA OR “acquired immunodeficiency syndrome*” OR “acquired immune-deficiency syndrome*” OR “acquired immune deficiency syndrome*” OR AIDS OR “human immunodeficiency virus*” OR HIV OR HIV1 OR HIV-1 OR HIV2 OR HIV-2 ) | 79,703 |
| 2 | Body Image | ( DE "Body Image" OR DE "Body Dissatisfaction" OR DE "Body Esteem") OR TI ( “body image*” OR body-image OR “body integrity” OR “body schema*” OR “body representation*” OR “body dissatisfaction” OR “body satisfaction” OR “body esteem” OR “body self-esteem” OR “body appreciation” OR “body shame” OR “body preoccupation” OR “body discomfort” OR “body perception” OR “body insecurity” OR “body acceptance” OR “body confidence” OR “body concern*” OR “body attitude*” OR “body awareness” OR “body dysphoria” OR “body dysmorph*” OR “body distortion” OR “body ideal” OR self-image OR self-concept OR self-representation OR “appearance evaluation” OR (appearance N3 satisfaction) OR (appearance N3 dissatisfaction) OR “appearance anxiety” OR “appearance concern*” OR “appearance-related anxiety” OR “appearance-related concern*” ) OR AB ( “body image*” OR body-image OR “body integrity” OR “body schema*” OR “body representation*” OR “body dissatisfaction” OR “body satisfaction” OR “body esteem” OR “body self-esteem” OR “body appreciation” OR “body shame” OR “body preoccupation” OR “body discomfort” OR “body perception” OR “body insecurity” OR “body acceptance” OR “body confidence” OR “body concern*” OR “body attitude*” OR “body awareness” OR “body dysphoria” OR “body dysmorph*” OR “body distortion” OR “body ideal” OR self-image OR self-concept OR self-representation OR “appearance evaluation” OR (appearance N3 satisfaction) OR (appearance N3 dissatisfaction) OR “appearance anxiety” OR “appearance concern*” OR “appearance-related anxiety” OR “appearance-related concern*” ) | 60,382 |
| 3 |  | #1 AND #2 | 476 |
| 4 | Language | #3 AND LA English | 445 |
| 5 | Date | #4 AND PY 2000-2024 | 350 |

( ( DE "AIDS" OR DE "HIV" ) OR TI ( “persons living with AIDS” OR “people living with AIDS” OR “persons living with HIV” OR “people living with HIV” OR PLHIV OR PLWH OR PLWHA OR “acquired immunodeficiency syndrome*” OR “acquired immune-deficiency syndrome*” OR “acquired immune deficiency syndrome*” OR AIDS OR “human immunodeficiency virus*” OR HIV OR HIV1 OR HIV-1 OR HIV2 OR HIV-2 ) OR AB ( “persons living with AIDS” OR “people living with AIDS” OR “persons living with HIV” OR “people living with HIV” OR PLHIV OR PLWH OR PLWHA OR “acquired immunodeficiency syndrome*” OR “acquired immune-deficiency syndrome*” OR “acquired immune deficiency syndrome*” OR AIDS OR “human immunodeficiency virus*” OR HIV OR HIV1 OR HIV-1 OR HIV2 OR HIV-2 ) ) AND ( ( DE "Body Image" OR DE "Body Dissatisfaction" OR DE "Body Esteem") OR TI ( “body image*” OR body-image OR “body integrity” OR “body schema*” OR “body representation*” OR “body dissatisfaction” OR “body satisfaction” OR “body esteem” OR “body self-esteem” OR “body appreciation” OR “body shame” OR “body preoccupation” OR “body discomfort” OR “body perception” OR “body insecurity” OR “body acceptance” OR “body confidence” OR “body concern*” OR “body attitude*” OR “body awareness” OR “body dysphoria” OR “body dysmorph*” OR “body distortion” OR “body ideal” OR self-image OR self-concept OR self-representation OR “appearance evaluation” OR (appearance N3 satisfaction) OR (appearance N3 dissatisfaction) OR “appearance anxiety” OR “appearance concern*” OR “appearance-related anxiety” OR “appearance-related concern*” ) OR AB ( “body image*” OR body-image OR “body integrity” OR “body schema*” OR “body representation*” OR “body dissatisfaction” OR “body satisfaction” OR “body esteem” OR “body self-esteem” OR “body appreciation” OR “body shame” OR “body preoccupation” OR “body discomfort” OR “body perception” OR “body insecurity” OR “body acceptance” OR “body confidence” OR “body concern*” OR “body attitude*” OR “body awareness” OR “body dysphoria” OR “body dysmorph*” OR “body distortion” OR “body ideal” OR self-image OR self-concept OR self-representation OR “appearance evaluation” OR (appearance N3 satisfaction) OR (appearance N3 dissatisfaction) OR “appearance anxiety” OR “appearance concern*” OR “appearance-related anxiety” OR “appearance-related concern*” ) ) AND LA English AND PY 2000-2024

**Supplementary Table 1d.** *Search strategy and search string used in* ***Embase****.*

| **Search #** | **Concept** | **Query** | **Results** |
| --- | --- | --- | --- |
| 1 | PLWH | 'aids patient'/exp OR 'human immunodeficiency virus infected patient'/exp OR 'acquired immune deficiency syndrome'/de OR 'human immunodeficiency virus'/exp OR 'persons living with aids':ab,ti OR 'people living with aids':ab,ti OR 'persons living with hiv':ab,ti OR 'people living with hiv':ab,ti OR plhiv:ab,ti OR plwh:ab,ti OR plwha:ab,ti OR 'acquired immunodeficiency syndrome*':ab,ti OR 'acquired immune-deficiency syndrome*':ab,ti OR 'acquired immune deficiency syndrome*':ab,ti OR aids:ab,ti OR 'human immunodeficiency virus*':ab,ti OR hiv:ab,ti OR hiv1:ab,ti OR 'hiv 1':ab,ti OR hiv2:ab,ti OR 'hiv 2':ab,ti | 661,086 |
| 2 | Body Image | 'body image'/exp OR 'body representation'/exp OR 'body satisfaction'/exp OR 'body esteem'/exp OR 'body appreciation'/exp OR 'body image*':ab,ti OR 'body integrity':ab,ti OR 'body schema*':ab,ti OR 'body representation*':ab,ti OR 'body dissatisfaction':ab,ti OR 'body satisfaction':ab,ti OR 'body esteem':ab,ti OR 'body self-esteem':ab,ti OR 'body appreciation':ab,ti OR 'body shame':ab,ti OR 'body preoccupation':ab,ti OR 'body discomfort':ab,ti OR 'body perception':ab,ti OR 'body insecurity':ab,ti OR 'body acceptance':ab,ti OR 'body confidence':ab,ti OR 'body concern*':ab,ti OR 'body attitude*':ab,ti OR 'body awareness':ab,ti OR 'body dysphoria':ab,ti OR 'body dysmorph*':ab,ti OR 'body distortion':ab,ti OR 'body ideal':ab,ti OR 'self image':ab,ti OR 'self concept':ab,ti OR 'self representation':ab,ti OR 'appearance evaluation':ab,ti OR (appearance NEAR/3 satisfaction) OR (appearance NEAR/3 dissatisfaction) OR 'appearance anxiety':ab,ti OR 'appearance concern*':ab,ti OR 'appearance-related anxiety':ab,ti OR 'appearance-related concern*':ab,ti | 54,545 |
| 3 |  | #1 AND #2 | 689 |
| 4 | Language | #3 AND [english]/lim | 663 |
| 5 | Date | #4 AND [2000-2024]/py | 612 |

('aids patient'/exp OR 'human immunodeficiency virus infected patient'/exp OR 'acquired immune deficiency syndrome'/de OR 'human immunodeficiency virus'/exp OR 'persons living with aids':ab,ti OR 'people living with aids':ab,ti OR 'persons living with hiv':ab,ti OR 'people living with hiv':ab,ti OR plhiv:ab,ti OR plwh:ab,ti OR plwha:ab,ti OR 'acquired immunodeficiency syndrome*':ab,ti OR 'acquired immune-deficiency syndrome*':ab,ti OR 'acquired immune deficiency syndrome*':ab,ti OR aids:ab,ti OR 'human immunodeficiency virus*':ab,ti OR hiv:ab,ti OR hiv1:ab,ti OR 'hiv 1':ab,ti OR hiv2:ab,ti OR 'hiv 2':ab,ti) AND ('body image'/exp OR 'body representation'/exp OR 'body satisfaction'/exp OR 'body esteem'/exp OR 'body appreciation'/exp OR 'body image*':ab,ti OR 'body integrity':ab,ti OR 'body schema*':ab,ti OR 'body representation*':ab,ti OR 'body dissatisfaction':ab,ti OR 'body satisfaction':ab,ti OR 'body esteem':ab,ti OR 'body self-esteem':ab,ti OR 'body appreciation':ab,ti OR 'body shame':ab,ti OR 'body preoccupation':ab,ti OR 'body discomfort':ab,ti OR 'body perception':ab,ti OR 'body insecurity':ab,ti OR 'body acceptance':ab,ti OR 'body confidence':ab,ti OR 'body concern*':ab,ti OR 'body attitude*':ab,ti OR 'body awareness':ab,ti OR 'body dysphoria':ab,ti OR 'body dysmorph*':ab,ti OR 'body distortion':ab,ti OR 'body ideal':ab,ti OR 'self image':ab,ti OR 'self concept':ab,ti OR 'self representation':ab,ti OR 'appearance evaluation':ab,ti OR (appearance NEAR/3 satisfaction) OR (appearance NEAR/3 dissatisfaction) OR 'appearance anxiety':ab,ti OR 'appearance concern*':ab,ti OR 'appearance-related anxiety':ab,ti OR 'appearance-related concern*':ab,ti) AND [english]/lim AND [2000-2024]/py

**Supplementary Table 1e.** *Search strategy and search string used in* ***Web of Science****.*

| **Search #** | **Concept** | **Query** | **Results** |
| --- | --- | --- | --- |
| 1 | PLWH | (TI=(“persons living with AIDS” OR “people living with AIDS” OR “persons living with HIV” OR “people living with HIV” OR PLHIV OR PLWH OR PLWHA OR “acquired immunodeficiency syndrome*” OR “acquired immune-deficiency syndrome*” OR “acquired immune deficiency syndrome*” OR AIDS OR “human immunodeficiency virus*” OR HIV OR HIV1 OR HIV-1 OR HIV2 OR HIV-2 )) OR AB=(“persons living with AIDS” OR “people living with AIDS” OR “persons living with HIV” OR “people living with HIV” OR PLHIV OR PLWH OR PLWHA OR “acquired immunodeficiency syndrome*” OR “acquired immune-deficiency syndrome*” OR “acquired immune deficiency syndrome*” OR AIDS OR “human immunodeficiency virus*” OR HIV OR HIV1 OR HIV-1 OR HIV2 OR HIV-2 ) | 1,077,196 |
| 2 | Body Image | (TI=(“body image*” OR body-image OR “body integrity” OR “body schema*” OR “body representation*” OR “body dissatisfaction” OR “body satisfaction” OR “body esteem” OR “body self-esteem” OR “body appreciation” OR “body shame” OR “body preoccupation” OR “body discomfort” OR “body perception” OR “body insecurity” OR “body acceptance” OR “body confidence” OR “body concern*” OR “body attitude*” OR “body awareness” OR “body dysphoria” OR “body dysmorph*” OR “body distortion” OR “body ideal” OR self-image OR self-concept OR self-representation OR “appearance evaluation” OR (appearance NEAR/3 satisfaction) OR (appearance NEAR/3 dissatisfaction) OR “appearance anxiety” OR “appearance concern*” OR “appearance-related anxiety” OR “appearance-related concern*”)) OR AB=(“body image*” OR body-image OR “body integrity” OR “body schema*” OR “body representation*” OR “body dissatisfaction” OR “body satisfaction” OR “body esteem” OR “body self-esteem” OR “body appreciation” OR “body shame” OR “body preoccupation” OR “body discomfort” OR “body perception” OR “body insecurity” OR “body acceptance” OR “body confidence” OR “body concern*” OR “body attitude*” OR “body awareness” OR “body dysphoria” OR “body dysmorph*” OR “body distortion” OR “body ideal” OR self-image OR self-concept OR self-representation OR “appearance evaluation” OR (appearance NEAR/3 satisfaction) OR (appearance NEAR/3 dissatisfaction) OR “appearance anxiety” OR “appearance concern*” OR “appearance-related anxiety” OR “appearance-related concern*”) | 58,589 |
| 3 |  | #1 AND #2 | 879 |
| 4 | Language | #3 AND (LA=English) | 841 |
| 5 | Date | #4 AND (PY=(2000-2024)) | 779 |

((TI=(“persons living with AIDS” OR “people living with AIDS” OR “persons living with HIV” OR “people living with HIV” OR PLHIV OR PLWH OR PLWHA OR “acquired immunodeficiency syndrome*” OR “acquired immune-deficiency syndrome*” OR “acquired immune deficiency syndrome*” OR AIDS OR “human immunodeficiency virus*” OR HIV OR HIV1 OR HIV-1 OR HIV2 OR HIV-2 )) OR AB=(“persons living with AIDS” OR “people living with AIDS” OR “persons living with HIV” OR “people living with HIV” OR PLHIV OR PLWH OR PLWHA OR “acquired immunodeficiency syndrome*” OR “acquired immune-deficiency syndrome*” OR “acquired immune deficiency syndrome*” OR AIDS OR “human immunodeficiency virus*” OR HIV OR HIV1 OR HIV-1 OR HIV2 OR HIV-2 )) AND ((TI=(“body image*” OR body-image OR “body integrity” OR “body schema*” OR “body representation*” OR “body dissatisfaction” OR “body satisfaction” OR “body esteem” OR “body self-esteem” OR “body appreciation” OR “body shame” OR “body preoccupation” OR “body discomfort” OR “body perception” OR “body insecurity” OR “body acceptance” OR “body confidence” OR “body concern*” OR “body attitude*” OR “body awareness” OR “body dysphoria” OR “body dysmorph*” OR “body distortion” OR “body ideal” OR self-image OR self-concept OR self-representation OR “appearance evaluation” OR (appearance NEAR/3 satisfaction) OR (appearance NEAR/3 dissatisfaction) OR “appearance anxiety” OR “appearance concern*” OR “appearance-related anxiety” OR “appearance-related concern*”)) OR AB=(“body image*” OR body-image OR “body integrity” OR “body schema*” OR “body representation*” OR “body dissatisfaction” OR “body satisfaction” OR “body esteem” OR “body self-esteem” OR “body appreciation” OR “body shame” OR “body preoccupation” OR “body discomfort” OR “body perception” OR “body insecurity” OR “body acceptance” OR “body confidence” OR “body concern*” OR “body attitude*” OR “body awareness” OR “body dysphoria” OR “body dysmorph*” OR “body distortion” OR “body ideal” OR self-image OR self-concept OR self-representation OR “appearance evaluation” OR (appearance NEAR/3 satisfaction) OR (appearance NEAR/3 dissatisfaction) OR “appearance anxiety” OR “appearance concern*” OR “appearance-related anxiety” OR “appearance-related concern*”)) AND (LA=English) AND (PY=(2000-2024))
